# Parameter inference for stochastic single-cell dynamics from lineage tree data

**DOI:** 10.1186/s12918-017-0425-1

**Published:** 2017-04-26

**Authors:** Irena Kuzmanovska, Andreas Milias-Argeitis, Jan Mikelson, Christoph Zechner, Mustafa Khammash

**Affiliations:** 1 Department of Biosystems Science and Engineering, ETH Zurich, Mattenstrasse 26, Basel, 4058 Switzerland; 2 Groningen Biomolecular Sciences and Biotechnology, University of Groningen, Nijenborgh 4, Groningen, 9747 AG Netherlands; 3 Max Planck Institute of Molecular Cell Biology and Genetics and Center for Systems Biology, Pfotenhauerstrasse 108, Dresden, 01307 Germany

**Keywords:** Parameter inference, Cell lineages, Single cell, Stochastic systems, Monte Carlo methods

## Abstract

**Background:**

With the advance of experimental techniques such as time-lapse fluorescence microscopy, the availability of single-cell trajectory data has vastly increased, and so has the demand for computational methods suitable for parameter inference with this type of data. Most of currently available methods treat single-cell trajectories independently, ignoring the mother-daughter relationships and the information provided by the population structure. However, this information is essential if a process of interest happens at cell division, or if it evolves slowly compared to the duration of the cell cycle.

**Results:**

In this work, we propose a Bayesian framework for parameter inference on single-cell time-lapse data from lineage trees. Our method relies on a combination of Sequential Monte Carlo for approximating the parameter likelihood function and Markov Chain Monte Carlo for parameter exploration. We demonstrate our inference framework on two simple examples in which the lineage tree information is crucial: one in which the cell phenotype can only switch at cell division and another where the cell state fluctuates slowly over timescales that extend well beyond the cell-cycle duration.

**Conclusion:**

There exist several examples of biological processes, such as stem cell fate decisions or epigenetically controlled phase variation in bacteria, where the cell ancestry is expected to contain important information about the underlying system dynamics. Parameter inference methods that discard this information are expected to perform poorly for such type of processes. Our method provides a simple and computationally efficient way to take into account single-cell lineage tree data for the purpose of parameter inference and serves as a starting point for the development of more sophisticated and powerful approaches in the future.

**Electronic supplementary material:**

The online version of this article (doi:10.1186/s12918-017-0425-1) contains supplementary material, which is available to authorized users.

## Background

Biochemical processes in isogenic cells exhibit substantial heterogeneity [1, 2]. Understanding the latter demands experimental techniques that can resolve such processes at the single-cell level. In contrast to bulk measurements, these techniques provide not only access to the average behavior of intracellular dynamics, but also its variability across cells and over time. Most single-cell techniques, however, reveal only very few components simultaneously that are often multiple steps away from the actual quantities of interest. The dynamics of a promoter, for instance, may not be accessible directly, but only indirectly through a fluorescent reporter that is expressed upon activation of this promoter [3]. Statistical inference in combination with mathematical models provide a means to reconstruct inaccessible parameters from available measurements, making them instrumental for studying biochemical processes based on single-cell data.

How such inference can be performed depends strongly on the way the data has been collected: flow cytometry measurements, for instance, reveal fluorescence values across a population but individual cells cannot be tracked over time. Consequently, measurements at two different time instances are considered statistically independent. Time-lapse microscopy techniques permit tracking of single-cell trajectories over the duration of a whole experiment [4], which in turn provides a handle also on the temporal correlation of the underlying process. This additional degree of information can dramatically improve the inference of unknown process parameters [5].

Most existing inference approaches consider single-cell trajectories to be statistically independent of each other [3, 5–7]. This way, however, important information stemming from the ancestry of a cell is lost: shortly after cell division, for example, two daughter cells are likely to exhibit substantial correlations, which cannot be captured by a model that assumes independence among cells. This can yield incomplete and biased results, especially when the time scale of the process under study is slow compared with the cell cycle duration.

In addition, stochastic processes of interest such as epigenetically regulated phase variation in bacteria are often driven by DNA replication just before cell division. Examples in this category are the regulation of *agn43* [8, 9] and *Pap* [10, 11] systems in *E.coli*, and the glucosyltransferase (*gtr*) gene cluster in *Salmonella* [12]. Due to the non-reversibility of the epigenetic modifications, gene replication (and consequently cell division) is crucial for phase variation to happen. Cell lineage information has to be therefore taken into account in single-cell studies of these systems.

Until recently, there existed little work on statistical inference using tree-based single-cell data. In [13], the authors proposed a method for parameter inference from single-cell trajectories based on Approximate Bayesian Computation (ABC). Their approach is applicable to tree-structured data as well, although it requires all trajectories to have the same length and sampling resolution. In [14] the authors proposed an observer-based method for state and parameter estimation in stochastic chemical reaction networks, which is also able to handle lineage tree data. However, its applicability is limited to small systems since it requires the full probability distributions from the solution of the chemical master equation. Another alternative was proposed in [15], which presented an inference algorithm for Hidden Markov Trees using variational Bayesian Expectation Maximization. This class of models is similar to the one considered here, but cannot incorporate dynamic readouts or dynamically evolving single-cell states.

In more recent work, the authors of [16] presented a method for inferring transition dynamics from cell lineages that is best suited to slowly evolving cell states (such as in the case of stem cell lineages) and makes use of end-point smFISH measurements for each cell. Finally, Feigelman et al. [17] proposed a method for exact Bayesian parameter inference from cell lineage data that uses particle filtering to approximate the full joint state and parameter posterior distribution. The method was successfully applied to a stochastic gene expression system that is critical for stem cell differentiation and clearly demonstrated the strengths of lineage-based inference. On the downside, the computational burden of the method seems to be substantial, while particle degeneracy may arise when trees longer than just a few generations are used because of the way particle sampling and reweighing are carried out.

In this work, we propose an approximate Bayesian parameter inference framework for lineage tree data. The method relies on a combination of Sequential Monte Carlo for likelihood approximation and pseudo-marginal Markov chain Monte Carlo for parameter sampling. To achieve scalability of our method with the number of generations, we make use of a plausible simplifying assumption in the likelihood decomposition which is shown to work well in practice. In contrast to [17], our method allows efficient likelihood calculation and smaller particle degeneracy with increasing tree lengths, which allows us to extract information out of longer lineages. Furthermore, parameter sampling and likelihood approximation are carried out separately from each other, which permits the use of more powerful samplers (such as Population Monte Carlo [18] or Nested Sampling [19]) for the efficient exploration of high-dimensional parameter spaces.

The rest of the manuscript is structured as follows: in ‘Methods’ section we give a mathematical description of the inference problem and the class of models we consider and we present a detailed description of our method. In ‘Results and discussion’ section we demonstrate the application of our method to two different example models and in ‘Conclusions’ section we give some concluding remarks.

## Methods

### The model class

**Fig. 1:**
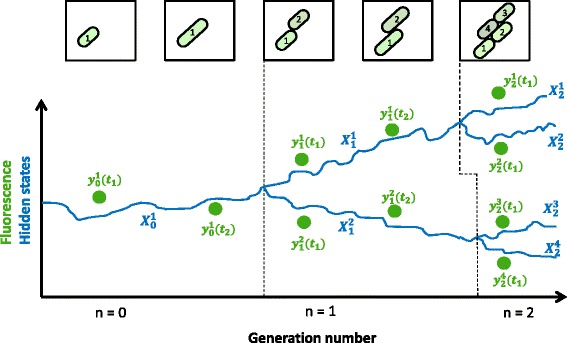
Graphical illustration of the observed and unobserved system dynamics in the lineage tree setting. A hypothetical time-lapse microscopy experiment, in which time-lapse microscopy images of a growing *E.coli* colony are obtained. The strain contains a fluorescent reporter gene. After the cells are segmented and tracked, the fluorescence intensity in each of them can be extracted, giving rise to a fluorescence lineage dataset (*green dots*). The continuous *blue curve* represents the unobserved state trajectory of each individual cell. Subscripts on measurements and states denote the generation number, while *superscripts* index the cells of each generation

To introduce the inference problem and the class of models considered here, we refer to the illustration in Fig. 1. Let us consider an intracellular biochemical process of interest modeled by a continuous-time dynamical system *S*. The system behavior within each cell can be monitored with the help of a dynamic readout, such as the abundance of a fluorescent reporter protein. Through time-lapse microscopy, we assume that a growing population of single cells and their progeny can be tracked over time and measured at multiple time points (green dots in Fig. 1), giving rise to a hierarchical tree data structure that describes the time evolution of the population.

We assume that each tree starts with a single mother at generation 0 and that the population is followed until the final generation *N*. Without loss of generality we assume that a single mother always gives rise to two daughter cells after division, leading to 2^*n*^ cells at generation *n*. The system *S* describes the evolution of a set of internal states *x* (schematically represented by blue curve in Fig. 1). These states can be accessed indirectly at discrete time points through experimental techniques yielding a corresponding readout *y*. Each cell is assigned a separate time index and a separate time of division, *T*, which can either be assumed known from the single-cell tracking data, or be inferred based on these data. We denote by ***X*** the whole trajectory {*x*(*t*),*t*∈[0,*T*]} from the time of birth of a cell (at *t*=0) until its division (at *t*=*T*). The dynamics of *S* may evolve on a continuous, discrete or hybrid space, and similarly be stochastic, deterministic, or involve components of both types. In any case, we assume that *S* depends on a set of parameters *Θ*, which are either assumed to be the same across the population or allowed to vary within the population according to a population-wide distribution.

From this point on, we will distinguish each cell by its generation number, *n*, and an index *i*, that ranges from 1 to 2^*n*^ (refer to Fig. 1). The *i* ^*th*^ cell of the *n* ^*th*^ generation gives rise to two daughters, indexed by 2*i*−1 and 2*i*, in generation *n*+1. Henceforth, all quantities related to a certain cell in a given lineage will be indexed by these two numbers.

Following this notation, we denote by $\boldsymbol {X}_{n}^{i}$ the state trajectory of the *i* ^*th*^ cell in the *n* ^*th*^ generation; that is,

$$\boldsymbol{X}_{n}^{i}:= \{x_{n}^{i}(t),t\in[0,T_{n}^{i}]\}. $$

The state trajectories of the daughters originating from a mother cell with state trajectory $\boldsymbol {X}_{n}^{i}$ will therefore be denoted by $\boldsymbol {X}_{n+1}^{2i-1}$ and $\boldsymbol {X}_{n+1}^{2i}$ respectively. The corresponding discrete set of measurements associated with $\boldsymbol {X}_{n}^{i}$ is denoted by $\boldsymbol {Y}_{n}^{i}$. More specifically,

$$\boldsymbol{Y}_{n}^{i}:=\{y_{n}^{i}(t_{n,k}^{i}),k=1,\dots,K_{n}^{i})\}. $$

This notation reflects the fact that the *i* ^*th*^ cell of the *n* ^*th*^ generation is observed at a total number of $K_{n}^{i}$ time points (each denoted by $t_{n,k}^{i}$) during its lifetime, and that the number and location of observation time points will in general be different for every cell.

We will further denote by $P(x_{n+1}^{2i-1}(0),x_{n+1}^{2i}(0)|x_{n}^{i}(T_{n}^{i}), \Theta \!)$ the distribution of the daughter initial conditions given the state of the mother just before division, and call this the *transition probability* from one generation to the next. It is reasonable to assume that, once their respective initial conditions are determined based on their mother cell, the two daughters evolve independently of each other. As defined above, the transition probability mechanism may itself contain unknown parameters that need to be estimated from the data.

### The inference problem

Our goal is to infer the posterior distribution of *Θ* given: 1. the set of measured cellular readouts over the whole lineage, 2. our prior knowledge about *Θ* encoded in a prior distribution *π*(*Θ*) and 3. a measurement noise model that describes the likelihood of observing $y_{n}^{i}(t)$ given $x_{n}^{i}(t)$ (possibly also depending explicitly on unknown parameters contained in *Θ*). The latter is given by the density $f(y_{n}^{i}(t)|x_{n}^{i}(t),\Theta)$. With this measurement model, and assuming that measurements at individual time points are independent from each other, the likelihood of the whole measurement set for a single cell can be defined as

$$P(\boldsymbol{Y}_{n}^{i} \mid \boldsymbol{X}_{n}^{i},\Theta)=\prod\limits_{k=1}^{K_{n}^{i}} f\left(y_{n}^{i}\left(t_{n,k}^{i})|x_{n}^{i}(t_{n,k}^{i}\right),\Theta\right). $$

Setting

$$\boldsymbol{X}_{tree}:=\{\boldsymbol{X}_{n}^{i},i=1,\dots,2^{n},~n=0,\dots,N\} $$

and

$$\boldsymbol{Y}_{tree}:=\{\boldsymbol{Y}_{n}^{i},i=1,\dots,2^{n},~n=0,\dots,N\}, $$

the joint distribution over states and measurements over a tree starting from a single individual can be written as

1 $$ { \begin{aligned} P(\boldsymbol{X}_{tree},\boldsymbol{Y}_{tree} \mid \Theta)&=P\left(\boldsymbol{X}_{0}^{1} \mid \pi_{x}\left(X_{0}^{1}\right)\right)P\left(\boldsymbol{Y}_{0}^{1} \mid \boldsymbol{X}_{0}^{1},\Theta\right) \\ &\quad\times \prod\limits_{n=1}^{N} \left[\! \left({\prod\limits_{i=1}^{2^{n}}} P\left(\boldsymbol{X}_{n}^{2i-1},\boldsymbol{X}_{n}^{2i} \mid \boldsymbol{X}_{n-1}^{i},\Theta\right) \right)\right.\\ &\left.\qquad\qquad\times\left({\prod\limits_{i=1 }^{2^{n}}} P\left(\boldsymbol{Y}_{n}^{i} \mid \boldsymbol{X}_{n}^{i},\Theta\right) \right) \right], \end{aligned}}  $$

where $\pi _{x}(X_{0}^{1})$ is the initial distribution of $x_{0}^{1}(0)$. The likelihood of the measured outputs given *Θ* can therefore by obtained by marginalization of (1) over all possible unobserved states:

2 $$ P(\boldsymbol{Y}_{tree} \mid \Theta)=\int P(\boldsymbol{X}_{tree},\boldsymbol{Y}_{tree} \mid \Theta)d\boldsymbol{X}_{tree}.  $$

As can be seen from the above equations, an additional difficulty of our inference problem in comparison to inference based on independent cell trajectories, is the fact that the likelihood *P*(***X***_*tree*_,***Y***_*tree*_∣*Θ*) does not factorize over the readouts of individual cells, since the tree structure of the population introduces dependencies among the observations coming from different generations. The dependencies are generated through the unobserved state dynamics, which must therefore be taken into account.

Moreover, due to the dependencies introduced by the tree structure of the population, the integral in (2) is analytically intractable already for very simple state dynamics and its numerical evaluation scales exponentially with the number of generations in the tree. To address these difficulties, we employ a sequential Monte Carlo (SMC) scheme as described below to approximate the marginal likelihood (2).

### Recursive likelihood and state posterior propagation

The joint likelihood over states and observations given by (1) can be recursively computed, for example by first iterating over generations and then over the individuals of each generation. However, the same cannot be immediately said for (2), where the marginalization complicates the calculation. Here we propose an iterative calculation of this likelihood that again proceeds sequentially through the tree generations and the daughter pairs of each generation. The dependencies between different daughter pairs of the same generation add to the complexity of the numerical approximation of the likelihood, but, as we will see at the end of the section, this computation can be sped up considerably by making a reasonable simplifying approximation.

Before we derive the exact formulas, we need some additional notation. Let

$$\boldsymbol{Y}^{1,\dots,2^{n}}_{n}:={\left\{\boldsymbol{Y}^{1}_{n},\dots,\boldsymbol{Y}^{2^{n}}_{n}\right\}} $$

denote the whole dataset of generation *n* and

$$\mathbb{Y}_{0:n}:={\left\{\boldsymbol{Y}^{1,\dots,2^{m}}_{m},m=0,\dots,n\right\}} $$

the dataset of all generations up to generation *n*. Similarly,

$$\boldsymbol{X}^{1,\dots,2^{n}}_{n}:={\left\{\boldsymbol{X}^{1}_{n},\dots,\boldsymbol{X}^{2^{n}}_{n}\right\}} $$

and

$$\mathbb{X}_{0:n}:={\left\{\boldsymbol{X}^{1,\dots,2^{m}}_{m},m=0,\dots,n\right\}}. $$

To arrive at the exact formula for the likelihood, we first break up the total likelihood over the generations as follows (the dependence on *Θ* is suppressed to simplify the notation):

$$P(\boldsymbol{Y}_{tree})=P(\boldsymbol{Y}^{1}_{0})\prod_{n=1}^{N} P\left(\boldsymbol{Y}^{1,\dots,2^{n}}_{n}|\mathbb{Y}_{0:n-1}\right). $$

Assume now that $P(\mathbb {Y}_{0:n})$ (i.e. the likelihood of the subtree consisting of the first *n* generations) is available, and so is $P(\mathbb {X}_{0:n}|\mathbb {Y}_{0:n})$ (the state posterior over the same subtree). Consider the first two individuals of generation *n*+1, with state trajectories $\boldsymbol {X}^{1}_{n+1}$ and $\boldsymbol {X}^{2}_{n+1}$, descending from the mother cell with state trajectory $\boldsymbol {X}^{1}_{n}$.

Adding the information of this daughter pair to the posterior of the previous generations, we get

3 $$ {\begin{aligned} &P\left(\boldsymbol{X}^{1}_{n+1},\boldsymbol{X}^{2}_{n+1},\mathbb{X}_{0:n}|\boldsymbol{Y}^{1}_{n+1},\boldsymbol{Y}^{2}_{n+1},\mathbb{Y}_{0:n}\right)\\ &=\frac{P\left(\boldsymbol{Y}^{1}_{n+1},\boldsymbol{Y}^{2}_{n+1},\mathbb{Y}_{0:n}|\boldsymbol{X}^{1}_{n+1},\boldsymbol{X}^{2}_{n+1},\mathbb{X}_{0:n}\right)P\left(\boldsymbol{X}^{1}_{n+1},\boldsymbol{X}^{2}_{n+1},\mathbb{X}_{0:n}\right)} {P\left(\boldsymbol{Y}^{1}_{n+1},\boldsymbol{Y}^{2}_{n+1},\mathbb{Y}_{0:n}\right)}\\ &=\frac{P\left(\boldsymbol{Y}^{1}_{n+1}|\boldsymbol{X}^{1}_{n+1}\right)P\left(\boldsymbol{Y}^{2}_{n+1}|\boldsymbol{X}^{2}_{n+1}\right)P\left(\boldsymbol{X}^{1}_{n+1},\boldsymbol{X}^{2}_{n+1}|\mathbb{X}_{0:n}\right)} {P\left(\boldsymbol{Y}^{1}_{n+1},\boldsymbol{Y}^{2}_{n+1}|\mathbb{Y}_{0:n}\right)}\\ &\quad \times \frac{P\left(\mathbb{Y}_{0:n}|\mathbb{X}_{0:n}\right)P\left(\mathbb{X}_{0:n}\right)}{P\left(\mathbb{Y}_{0:n}\right)}\\ &=\frac{P\left(\boldsymbol{Y}^{1}_{n+1}|\boldsymbol{X}^{1}_{n+1}\right)P\left(\boldsymbol{Y}^{2}_{n+1}|\boldsymbol{X}^{2}_{n+1}\right)P\!\left(\boldsymbol{X}^{1}_{n+1},\boldsymbol{X}^{2}_{n+1}|\boldsymbol{X}^{1}_{n}\right)} {P\left(\boldsymbol{Y}^{1}_{n+1},\boldsymbol{Y}^{2}_{n+1}|\mathbb{Y}_{0:n}\right)}P(\mathbb{X}_{0:n}|\mathbb{Y}_{0:n}). \end{aligned}}  $$

The denominator of Eq. (3) extends $P(\mathbb {Y}_{0:n})$ with the daughter pair of the next generation:

4 $$\begin{array}{*{20}l}  &P(\boldsymbol{Y}^{1}_{n+1},\boldsymbol{Y}^{2}_{n+1}|\mathbb{Y}_{0:n})=  \\ &\iiint \Big(P\left(\boldsymbol{Y}^{1}_{n+1},\boldsymbol{Y}^{2}_{n+1}|\boldsymbol{X}^{1}_{n+1},\boldsymbol{X}^{2}_{n+1}\right)\\ &\times P\left(\boldsymbol{X}^{1}_{n+1},\boldsymbol{X}^{2}_{n+1}|\boldsymbol{X}^{1}_{n}\right) d\boldsymbol{X}^{1}_{n+1}d\boldsymbol{X}^{2}_{n+1}\Big)  \\ &\times P(\boldsymbol{X}^{1}_{n}|\mathbb{Y}_{0:n})d\boldsymbol{X}^{1}_{n} \end{array} $$

Equations. (3) and (4) allow us to update the starting posterior and likelihood with the first daughter pair from generation *n*+1. However, to add the second daughter pair (cells 3 and 4 of generation *n*+1, descending from cell 2 of generation *n*), we need to take into account the information provided by the first pair. To see this, we proceed as above (3) to arrive at:

5 $$\begin{array}{*{20}l} &P\left(\boldsymbol{X}^{3,4}_{n+1},\boldsymbol{X}^{1,2}_{n+1},\mathbb{X}_{0:n}|\boldsymbol{Y}^{3,4}_{n+1},\boldsymbol{Y}^{1,2}_{n+1},\mathbb{Y}_{0:n}\right)  \\ &=P\left(\boldsymbol{X}^{1,2}_{n+1},\mathbb{X}_{0:n}|\boldsymbol{Y}^{1,2}_{n+1},\mathbb{Y}_{0:n}\right)\\ &\quad \times\frac{P\left(\boldsymbol{Y}^{3,4}_{n+1}|\boldsymbol{X}^{3,4}_{n+1}\right)P\left(\boldsymbol{X}^{3,4}_{n+1}|\boldsymbol{X}^{1,2}_{n+1},\mathbb{X}_{0:n}\right)} {P(\boldsymbol{Y}^{3,4}_{n+1}|\boldsymbol{Y}^{1,2}_{n+1},\mathbb{Y}_{0:n})}. \end{array} $$

The above expression can be simplified by noting that $P\big (\boldsymbol {X}^{3,4}_{n+1}|\boldsymbol {X}^{1,2}_{n+1},\mathbb {X}_{0:n}\big)=P\big (\boldsymbol {X}^{3,4}_{n+1}|\mathbb {X}_{0:n}\big)$, i.e. daughter pairs of the same generation are conditionally independent given the parent states. However, the term $P\left (\boldsymbol {X}^{1,2}_{n+1},\mathbb {X}_{0:n}|\boldsymbol {Y}^{1,2}_{n+1},\mathbb {Y}_{0:n}\right)$ implies that, by taking into account the measurements of the first daughter pair, our posterior belief about the *n*-th generation states also needs to be updated before proceeding to the next pair. This leads to the creation of dependencies between the tree branches and means that they cannot be treated independently of each other, a feature than can create computational difficulties when one attempts to approximate the joint posterior by simulation. We thus make the *simplifying assumption* that

6 $$\begin{array}{*{20}l}  &P(\boldsymbol{X}^{i}_{n}|\boldsymbol{Y}^{2i-1,2i}_{n+1},\mathbb{Y}_{0:n})\approx P(\boldsymbol{X}^{i}_{n}|\mathbb{Y}_{0:n}) \end{array} $$

In words, we assume that *the additional state information transferred from the measurement of a daughter pair at generation* *n*+1* to their corresponding mother at generation n is negligible in comparison to the information provided by the previous generations to the mother*. This is especially the case when frequent observations of a cell population are available. In such a setting, the state of the mother can already be well constrained by measurements of itself and its ancestors, making the additional information provided by the daughters less significant. In case of very sparse measurements which are only available for the daughter cells right after cell division it is not certain to what extent the assumption will hold, since these measurements will also carry information about the state of the mother cell. However, frequent observations of the cells in the lineage can be easily achieved with currently used time-lapse microscopy methods.

As we will show below, the aforementioned assumption allows us to treat each mother-daughters triplet within a generation independently from the rest. Continuing the analysis of the first two daughter pairs from above, we have that

7 $$\begin{array}{*{20}l} &P\left(\boldsymbol{X}^{1,2}_{n+1},\mathbb{X}_{0:n}|\boldsymbol{Y}^{1,2}_{n+1},\mathbb{Y}_{0:n}\right)\\ &= P\left(\boldsymbol{X}^{1,2}_{n+1}|\mathbb{X}_{0:n},\boldsymbol{Y}^{1,2}_{n+1},\mathbb{Y}_{0:n}\right) P\left(\mathbb{X}_{0:n}| \boldsymbol{Y}^{1,2}_{n+1},\mathbb{Y}_{0:n}\right)  \\ &\approx P\left(\boldsymbol{X}^{1,2}_{n+1}|\mathbb{X}_{0:n},\boldsymbol{Y}^{1,2}_{n+1},\mathbb{Y}_{0:n}\right) P\left(\mathbb{X}_{0:n}|\mathbb{Y}_{0:n}\right)  \\ &= P\left(\boldsymbol{X}^{1,2}_{n+1}|\mathbb{X}_{0:n},\boldsymbol{Y}^{1,2}_{n+1}\right) P\left(\mathbb{X}_{0:n}|\mathbb{Y}_{0:n}\right). \end{array} $$

This fact therefore leads to a simplification of the conditional likelihood, $P(\boldsymbol {Y}^{3,4}_{n+1}|\boldsymbol {Y}^{1,2}_{n+1},\mathbb {Y}_{0:n})$:

8 $$ { \begin{aligned} &P(\boldsymbol{Y}^{3,4}_{n+1}|\boldsymbol{Y}^{1,2}_{n +1},\mathbb{Y}_{0:n}) \\ &=\iiint \Big(P\big(\boldsymbol{Y}^{3,4}_{n+1}|\boldsymbol{X}^{3,4}_{n+1}\big)P\big(\boldsymbol{X}^{3,4}_{n+1}|\boldsymbol{X}^{2}_{n}\big)d\boldsymbol{X}^{3,4}_{n+1}\Big) \\ &\qquad \times P\big(\boldsymbol{X}^{1,2}_{n+1}|\boldsymbol{X}^{2}_{n},\boldsymbol{Y}^{1,2}_{n+1}\big)P\left(\boldsymbol{X}^{2}_{n}|\mathbb{Y}_{0:n}\right)d\boldsymbol{X}^{1,2}_{n+1}d\boldsymbol{X}^{2}_{n} \\ &=\iint \Big(P\big(\boldsymbol{Y}^{3,4}_{n+1}|\boldsymbol{X}^{3,4}_{n+1}\big)P\big(\boldsymbol{X}^{3,4}_{n+1}|\boldsymbol{X}^{2}_{n}\big)d\boldsymbol{X}^{3,4}_{n+1}\Big) P\left(\boldsymbol{X}^{2}_{n}|\mathbb{Y}_{0:n}\right)\\ &\qquad \times d\boldsymbol{X}^{2}_{n}= P\left(\boldsymbol{Y}^{3,4}_{n+1}|\mathbb{Y}_{0:n}\right), \end{aligned}}  $$

and the total likelihood of generation *n*+1 (conditioned on $\mathbb {Y}_{0:n}$) can be decomposed as a product of likelihoods over the individual daughter pairs.

Finally, the joint posterior (5) can be also decomposed as:

9 $$ {\begin{aligned} &P\left(\boldsymbol{X}^{3,4}_{n+1},\boldsymbol{X}^{1,2}_{n+1},\mathbb{X}_{0:n}|\boldsymbol{Y}^{3,4}_{n+1},\boldsymbol{Y}^{1,2}_{n+1},\mathbb{Y}_{0:n}\right)\\ &=P\left(\boldsymbol{X}^{1,2}_{n+1}|\boldsymbol{Y}^{1,2}_{n+1},\mathbb{X}_{0:n}\right)P\left(\boldsymbol{X}^{3,4}_{n+1}|\boldsymbol{Y}^{3,4}_{n+1},\mathbb{X}_{0:n}\right) P\left(\mathbb{X}_{0:n}|\mathbb{Y}_{0:n}\right). \end{aligned}}  $$

These facts will be put in use in the next section, where a sequential Monte Carlo algorithm for the approximation of the tree likelihood will be presented.

### Recursive likelihood approximation

Our SMC scheme is used to approximate *P*(***Y***_*tree*_∣*Θ*), i.e. the likelihood of a set of measurements over a tree starting from a single individual, given a set of parameters *Θ*, under the simplifying assumption presented above. Our algorithm uses this assumption to exploit the conditional independence structure of the tree dynamics it generates in order to break down the likelihood computation. More concretely, the idea is to start at the root of the tree (i.e., a single cell) and recursively propagate the data likelihood from one generation to the next, treating the mother-daughter triplets of each generation independently from each other. This can be understood as a generalization of recursive filtering for tree-structured data. To illustrate the idea better, we present the treatment of a single mother-daughter triplet in detail.

Given data up to generation *n*, assume that *L* samples (particles) from the already estimated posterior $P(x_{n}^{i}(T_{n}^{i}) \mid \mathbb {Y}_{0:n})$ of the *i*-th mother in the *n*-th generation are available. First, a pair of daughter cells is generated according to the transition probabilities $P\left (x_{n+1}^{2i-1}(0), x_{n+1}^{2i}(0) | x_{n}^{i}(T_{n}^{i}), \Theta \right)$ for each particle. Given the daughters’ initial conditions, we next simulate each daughter until its own division time and calculate the likelihoods $P\left (\boldsymbol {Y}_{n+1}^{2i-1} \mid \boldsymbol {X}_{n+1}^{2i-1,l}, \Theta \right)$ and $P\left (\boldsymbol {Y}_{n+1}^{2i} \mid \boldsymbol {X}_{n+1}^{2i,l},\Theta \right)$ for *l*=1,…,*L*.

By assigning to the *l*-th particle a weight

$$w_{n+1}^{i,l}=P\left(\boldsymbol{Y}_{n+1}^{2i-1} \mid \boldsymbol{X}_{n+1}^{2i-1,l}, \Theta\right)P\left(\boldsymbol{Y}_{n+1}^{2i} \mid \boldsymbol{X}_{n+1}^{2i,l},\Theta\right), $$

we next compute the marginal likelihood of the *i* ^*th*^ mother-daughter triplet by averaging the weights for all the particles:

$$P\left(\boldsymbol{Y}_{n+1}^{2i-1},\boldsymbol{Y}_{n+1}^{2i}|\Theta\right)=\frac{1}{L}\sum\limits_{l=1}^{L} w_{n+1}^{i,l}. $$

After normalizing the particle weights to sum up to one, we have obtained weighted samples from the posteriors $P(\boldsymbol {X}_{n+1}^{2i-1} \mid \boldsymbol {Y}_{n+1}^{2i-1},\Theta)$ and $P(\boldsymbol {X}_{n+1}^{2i} \mid \boldsymbol {Y}_{n+1}^{2i},\Theta)$. The samples are subsequently unweighted by resampling *L* particles from each posterior according to the normalized weights. These samples will serve as starting points for the daughters of the next generation. The same process is repeated for the rest of the *n* ^*th*^ generation mothers, before moving on to generation *n*+1. This very general procedure is summarized in Algorithm 1, in which for simplicity we assume $\boldsymbol {X}_{0}^{1}$ to be known.



**Notes**

1. To simplify the presentation of the algorithm, the state trajectory of the initial mother cell was assumed known. In practice it is not and the initial conditions for the particles $\{x_{0}^{1,l}(0)\}_{l=1}^{L}$ would be drawn from a prior distribution. Then, a classical filtering step [7, 20] can be employed to obtain the final particle conditions for the first mother.
2. The most computationally intensive step of the algorithm lies between lines 5-9, where the marginal likelihood of each mother-daughter triplet needs to be computed. Depending on the type of the unobserved state dynamics, accurate marginalization may require the use of very large particle numbers and greatly increase the computational cost of the algorithm. Typically, the situation is worse when the hidden state contains components driven by stochastic dynamics. This challenge has already been recognized and addressed in the literature, since it also appears in the parameter inference problem from independent single-cell trajectories [7, 21]. One can thus employ one of the several available alternatives at this step, such as sequential computation of the likelihood [7], or the use of approximating dynamics [7, 22, 23].
3. Since we assume that the measurements from individual trees are independent from each other, the joint likelihood of a dataset consisting of several trees is simply a product of the likelihood of the individual trees. The likelihoods of individual trees can be thus estimated in parallel. Moreover, looking at the algorithm structure for a single tree, the likelihood calculation can be parallelized at two levels: 1) the mother cells of a given generation can be treated independently of each other 2) individual particle calculations for a given mother-daughter triplet can be done in parallel.

### A pseudo-marginal MCMC sampler for parameter inference

The goal of Bayesian inference is to compute or approximate via sampling the posterior distribution of the parameters of the system *Θ*, *P*(*Θ*|***Y***_*tree*_)∝*P*(***Y***_*tree*_|*Θ*)*π*(*Θ*). To this end, we follow the “pseudo-marginal” MCMC approach [24], according to which a Markov chain Monte Carlo (MCMC) sampler makes use of the noisy marginal likelihood estimates provided by the SMC algorithm of the previous section to generate samples from the posterior of *Θ* (Algorithm 2).



It should be noted that the use of very noisy SMC estimates may considerably slow down the mixing of the sampler, since the chain may get trapped at a point with artificially large likelihood value. However, as the variance of the estimator decreases (e.g. through the use of larger particle sample sizes), it is expected that the mixing speed of our sampler will converge to that of a sampler with perfect (i.e. noiseless) marginal likelihood information.

## Results and discussion

In the following sections we will consider two possible examples for the dynamical system *S* and demonstrate the application of our inference method on these cases. We use the first example primarily to verify and characterize the performance of our algorithm and the second example to convincingly demonstrate its application on a more complex problem. In both examples, we assume that a cell is characterized by a discrete state, *x*_*d*_(*t*). Over time and across generations, cells stochastically adopt a certain *type* (which for example corresponds to cell phenotype) determined by *x*_*d*_(*t*). The cell type in turn determines the evolution of a continuous state vector, which may for example correspond to the immature and mature molecule types of a fluorescent reporter. In abstract terms, *x*_*d*_(*t*) may be thought of as the state of a gene, whose activity affects the cell phenotype.

In the first example, the discrete state dynamics is described by a generalized two-type branching process. According to this scenario, the type of a cell is fixed throughout its lifetime and may change only at cell division, since the types of the daughters depend probabilistically on the type of the mother. In the second example, the cell type may stochastically vary throughout the cell lifetime according to a two-state continuous-time Markov chain (CTMC), while the two daughters are assumed to inherit the type of the mother. To test the performance of our inference framework, we generated simulated datasets for the two example systems (Additional file 1: Figure S1) and used them to infer parameters of interest in each case. Details about some of the parameters used in the data generation process are provided in Additional file 1: Table S1. The results for each example system are summarized below.

### Example 1: a two-type branching process with dynamic readouts

**Fig. 2:**
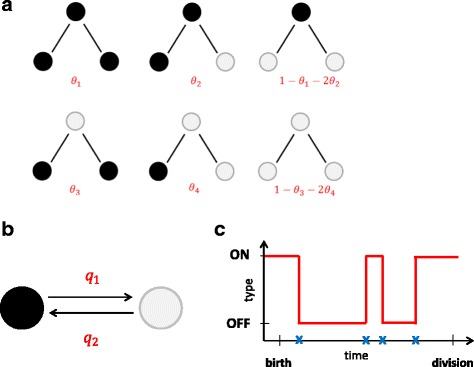
Graphical illustration of the two systems considered. **a** Model 1: All possible daughter pairs from a single mother and the corresponding probabilities of obtaining those pairs. The cells can be found in two possible states OFF (*black*) and ON (*white*). Note that the probability of the first daughter cell being OFF and the second ON is equal as the probability of the first daughter being ON and the second OFF. **b** Model 2: Cell types switch during the cell lifetime according to a two state continuous-time Markov chain with rates *q*_1_ and *q*_2_, **c** Model 2: An example of the evolution of a cell type during its lifetime. The *blue crosses* on the time axis indicate switching point from ON-OFF or vice versa

In this example, cells can adopt one of two possible types (ON or OFF) and maintain their type throughout their lifetime, which, for simplicity, we assume to be the same and equal to *T* for every cell. At cell division, the daughter cell types are determined based on the type of the mother cell, according to a set of transition probabilities, as illustrated in Fig. 2 a. In turn, the type of each cell is assumed to determine the production rate of a fluorescent reporter protein (such as GFP) which can then be observed using fluorescence time-lapse microscopy.

The state vector of each cell is thus defined as $x=\begin {bmatrix}x_{d}(t) &D(t)&F(t)\end {bmatrix}$, where *x*_*d*_(*t*) contains the cell type, while *D*(*t*) and *F*(*t*) correspond to the concentrations of the immature (dark) and mature (fluorescent) forms of the fluorescent reporter, respectively. Out of these, we assume that we can only obtain noisy measurements of *F*(*t*) at discrete points in time. Contrary to the cell type, the concentrations of the two reporter species are carried over from the mother to the daughters unchanged. That is, $D_{n+1}^{2i-1}(0) = D_{n}^{i}(T)$, $F_{n+1}^{2i-1}(0) = F_{n}^{i}(T)$ and similarly for the second daughter. This is a reasonable modeling assumption, given that a daughter cell has half the volume of the mother and receives roughly half of its protein content as well.

The fluorescent reporter dynamics of every cell evolves according to the following set of linear ODEs:

10 $$\begin{array}{*{20}l} \dot{D}(t) &= \alpha(x_{d}(t)) - \delta \cdot D(t) - m \cdot D(t) \end{array} $$

11 $$\begin{array}{*{20}l} \dot{F}(t)&= - \delta \cdot F(t) + m \cdot D(t), \end{array} $$

where *α*(*x*_*d*_(*t*)), *δ* and *m* are reporter protein production, dilution and maturation rates respectively. The production rate is determined by the cell type: for an OFF-type cell, *α*(*O* *F* *F*)=*α*_*OFF*_, while a cell of the ON type has *α*(*O* *N*)=*α*_*ON*_>*α*_*OFF*_.

As described above, we assume that noise-corrupted measurements proportional to the *F*(*t*) species are available at *M* points, *t*_1_,...,*t*_*M*_, during the life of every cell. The readout of a single cell at a given time is therefore assumed to be a scaled and noisy version of the *F*(*t*) concentration:

$$\begin{array}{*{20}l} &y(t_{m}) \sim \mathcal{N}(c \cdot F(t_{m}),\sigma^{2} \cdot F(t_{m})),  \end{array} $$

where the scaling constant *c* and the measurement variance *σ* ^2^ are known. The intuition behind this noise model is that a single concentration unit of mature GFP emits fluorescence which is normally distributed with mean *c* and variance *σ* ^2^. Therefore, for *F*(*t*) concentration units of mature GFP, the overall fluorescence emitted will be normally distributed with mean *c*·*F*(*t*) and variance *σ* ^2^·*F*(*t*). The issue of mapping from protein concentrations to fluorescence intensities is still not well-established in the literature, but several possible approaches have been proposed. The method presented in [25] exploits the deviations in daughter cell fluorescence levels from the average at each cell division. An alternative approach, suggested in [5] is based on recording a calibration curve with several proteins of known abundance fused to the same fluorescence tag.

Given that individual measurements for each cell are independent from each other, the expression for the likelihood $P(\boldsymbol {Y}_{n}^{i} \mid \boldsymbol {X}_{n}^{i}, \Theta)$, where $\boldsymbol {Y}_{n}^{i}=\{y_{n}^{i}(t_{m}),~m=1,\dots,M\}$, is given by

12 $$ P\left(\boldsymbol{Y}_{n}^{i} \mid \boldsymbol{X}_{n}^{i}\right) = \prod\limits_{m = 1}^{M} P\left(y_{n}^{i}(t_{m}) \mid F_{n}^{i}(t_{m})\right).  $$

Using this type of reporter measurements for every cell belonging to a fully observed tree, our goal is to infer the transition probabilities that govern cell type switching (*θ*_1_,…,*θ*_4_ in Fig. 2 a).

If the type of each cell was readily measurable, the use of simple maximum likelihood estimators for branching processes would suffice to obtain all the necessary discrete state statistics from a fully observed tree, making the use of the reporter model unnecessary. However, the intervening reporter maturation step, the slow dilution dynamics and the sparse, noisy sampling, make inference much more challenging and require the use of the sophisticated computational machinery presented in this work.

To test the performance of our algorithm on this system we simulated a synthetic dataset comprising of a single tree seven generations long (Additional file 1: Figure S1). The lifetime of each cell in the dataset was fixed at 30 min and the measurement interval was 5 min. We generated the daughter types according to the transition probabilities shown in Table 1. The rest of the parameters used for the data generation are summarized in Additional file 1: Table S1.

**Table 1:** Transition probabilities for the cell types considered in Example 1 and depicted on Fig. 2

		Daughter Types			
		(OFF,OFF)	(OFF,ON)	(ON,OFF)	(ON,ON)
Mother type	OFF	*θ*_1_=0.6	*θ*_2_=0.1	*θ*_2_	1−*θ*_1_−2*θ*_2_
	ON	*θ*_3_=0.1	*θ*_4_=0.05	*θ*_4_	1−*θ*_3_−2*θ*_4_

Note that due to symmetry, the second and third entries of each row are equal. Moreover, the values of the first and second entries in each row determine the rest of the entries, since every row sums to one. We therefore considered *θ*_1_, *θ*_2_, *θ*_3_ and *θ*_4_ as unknown.

We ran the pseudo-marginal MCMC sampler (Algorithm 2) to generate samples from the posterior distribution of *Θ*=[*θ*_1_ *θ*_2_ *θ*_3_ *θ*_4_]. The transition probabilities *θ*_1_ and *θ*_2_ were sampled with the help of a Dirichlet distribution (more details are given in Additional file 1) and similarly for *θ*_3_ and *θ*_4_. For all of the parameters we considered flat priors supported on the interval [0,1]. The initial values of *θ*_1_−*θ*_4_ were chosen to be all equal to 0.25, with the initial assumption that there are equal probabilities for all of the four possible transitions from a single mother. The number of SMC particles used for the inference procedure was 1000.

**Fig. 3:**
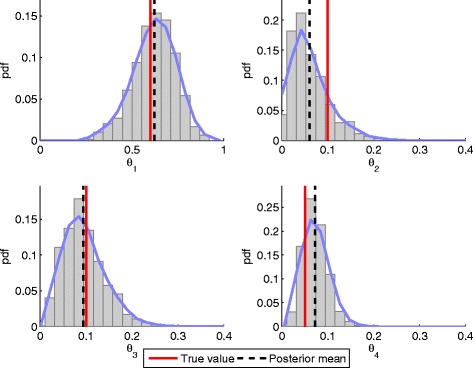
The proposed algorithm successfully infers parameters involved in the cell division process. Posterior distributions of the inferred parameters described in Example 1. The posteriors were obtained using 16,604 samples, after 2000 burn-in samples had been discarded. The *red vertical bar* is positioned at the true parameter value (the one used for data generation), while the *black dashed line* is positioned at the estimated posterior mean. The *blue curves* are obtained by smoothing of the normalized histograms of the samples

The estimated posterior distributions *P*(*Θ*|*Y*) based on 16604 MCMC samples are given in Fig. 3, where it can be clearly seen that the inferred posterior means (black dashed lines) are located close to the true parameter values (red lines). On Additional file 1: Figure S2 we can see that the sampler takes very few iterations to find the high-log-likelihood region. The movement of the chain is shown in Additional file 1: Figure S3, the autocorrelation of the samples is given in Additional file 1: Figure S4, while their pairwise scatter plots are given in Additional file 1: Figure S5. Sufficient number of independent samples (low autocorrelation) need to be obtained in order to be confident that they are representative of the true posterior distribution. To check this, we also thinned the chain by using every 10^*th*^ sample, after discarding the first 1000 burn-in samples. While the thinned chain exhibits much lower sample autocorrelation than the original chain (Additional file 1: Figure S6), the obtained posteriors in Additional file 1: Figure S7 are visually identical to those in Fig. 3. The raw data files generated by the sampler, which were used to generate some of the aforementioned figures, are given in Additional file 2.

**Fig. 4:**
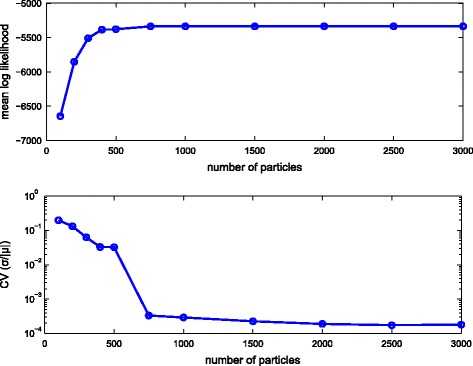
Convergence of the SMC likelihood estimator with increasing numbers of particles. Mean (*top*) and coefficient of variation (*bottom*) of the log-likelihood vs. number of particles used in the our likelihood estimation algorithm. To avoid negative values, the coefficient of variation was calculated by dividing the standard deviation by the absolute value of the mean, and plotted in log_10_ scale for better visualization. The results were based on 100 repetitions of the likelihood estimation for each particle count

To assess the variability of the likelihood SMC estimator as a function of the number of particles we estimated the log likelihood *P*(*Y*_*tree*_|*Θ*_*true*_) of the same dataset with different numbers of particles, given the true parameter values *Θ*_*true*_. As it can be seen on Fig. 4 (top), the average of 100 log-likelihood estimates converges as the particle number increases. With the increase of the number of particles the coefficient of variation of the log-likelihood calculations also drops quickly (Fig. 4 (bottom)). To avoid negative values we computed the coefficient of variation by dividing the standard deviation by the absolute value of the mean of the log-likelihood. We used the convergence properties of the estimator to get an initial sense of the order of magnitude for the particle number in the SMC algorithm. The particle number choice was refined empirically, based on the mixing and convergence behavior of the pseudo-marginal MCMC sampler. It can be seen in Fig. 4 that the estimator starts converging when around 750 particles are used. For the above-mentioned inference run we used 1000 particles.

To verify computationally that the simplifying assumption we employ in Eq. 6 does not lead to considerable bias in the estimated posteriors, we performed an inference run in which the assumption was not employed and the full likelihood was calculated using a particle filter based on the exact formulas presented above. The obtained posterior distributions (Additional file 1: Figure S7) are visually identical to the ones in Fig. 3, indicating that the simplifying assumption does not create significant bias of the inference results for the example considered here. The mean, variance and the median of the two sets of marginal posterior distributions of each inferred parameter are compared side-by-side in Additional file 1: Table S3. Their similarity indicates that by employing the approximation the basic features of the distributions are maintained.

### Example 2: stochastic cell type switching

In the second example, we assume that the cell type evolves according to a two-state CTMC with rates *q*_1_ and *q*_2_ for the OFF-to-ON and ON-to-OFF transitions respectively. At division, each daughter inherits the type of its mother (together with the reporter concentrations, as before), but subsequently evolves independently from other cells according to the CTMC dynamics, as shown on Fig. 2 b and c. In this case, during the cell lifetime the reporter production rate alternates between *α*_*ON*_ and *α*_*OFF*_ in accordance with the cell type.

Furthermore, to simulate a more biologically realistic scenario, we also incorporated extrinsic variability in our model by considering different GFP production rates for different cells. When a single cell was born, the GFP production rates *α*_*OFF*_ and *α*_*ON*_ were drawn in a correlated fashion from lognormal distribution with log-mean at $[\mu _{\alpha _{OFF}}~\mu _{\alpha _{ON}}]$. The value of the log-standard deviation of each marginal distribution *σ*_*ext*_ (henceforth referred to as ‘extrinsic noise’) was chosen to be 0.3. More concretely, the production rates were drawn by first drawing *z* from log$\mathcal {N}(1,\sigma _{ext}^{2})$ and then defining *α*_*OFF*_ and *α*_*ON*_ as described below. These production rates were subsequently used throughout the cell lifetime.

13 $$\begin{array}{*{20}l} & z \sim \log\mathcal{N}(1,\sigma_{ext}^{2}) \\  & \alpha_{OFF} = \mu_{\alpha_{OFF}} \cdot z \\  & \alpha_{ON} = \mu_{\alpha_{ON}} \cdot z  \end{array} $$

For the inference, the production rates were drawn at the beginning of the cell lifetime in a similar fashion for each particle.

Using the same type of reporter model dynamics and readouts as in the previous example, our goal in this case was to infer the CTMC transition rates *q*_1_ and *q*_2_ (Fig. 2 b), the production rate log-mean of the ON cells $\mu _{\alpha _{ON}}$, the dilution rate *δ*, the extrinsic noise *σ*_*ext*_ and the measurement variance *σ* ^2^, assuming the rest of the parameters to be known (i.e. $\Theta = \left [q_{1}~q_{2}~\mu _{\alpha _{ON}}~\delta ~\sigma _{ext}~\sigma ^{2}\right ]$).

While in the first example system the tree-structure information was essential for inference as the cell type can only change at cell division, it was not equally obvious that our method would outperform traditional inference on independent single-cell trajectories in this example system, where each daughter inherits the state of its mother and then evolves independently. To verify this, we additionally performed parameter inference (using the same MCMC sampler) by breaking up the tree into individual cell trajectories and considering each cell independently from the others (see Additional file 1), as is usually done in conventional inference based on single-cell data.

**Fig. 5:**
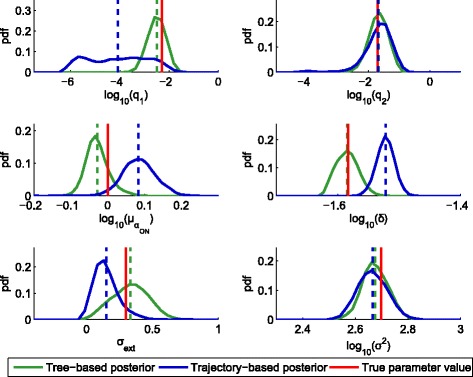
The proposed tree-based inference method outperforms the traditionally used inference on independent cell trajectories. Posterior distributions of the six unknown parameters presented in Example 2, obtained both with tree-based (*green*) and trajectory-based inference (*blue*). The *red bars* are positioned at the true parameter values (i.e. the ones used for data generation), while the *dashed lines* indicate the estimated posterior means. The curves are obtained by smoothing of the normalized histograms of the MCMC samples. One can clearly observe the bias in the parameter estimates when trajectory-based inference was used

To ensure that the MCMC chains converge to the same region of the parameter space, we performed several independent inference runs for each type of data by using the same sampler settings. After obtaining sufficiently long MCMC chains (Additional file 1: Figures S9 and S10) we thinned the chains as described in Additional file 1. The posterior distributions obtained from the thinned chains from a single tree-based and trajectory-based inference run are plotted and compared in Fig. 5. The posterior distribution sets obtained from the multiple independent MCMC runs are overlayed in Additional file 1: Figure S12. It can be easily seen that posterior distributions based on individual cell trajectories are in several cases biased, which indicates a potential disadvantage of the traditionally used trajectory-based inference. More details about the inference runs, including the prior distribution for the parameters and the proposal kernels used in the MCMC are given in Additional file 1, along with the running log-likelihoods, autocorrelation plots and the pairwise scatter plots of some of the samples. The raw data files produced by some of the MCMC runs (including all the accepted parameters and their corresponding log-likelihood values) are given in Additional file 2.

The advantage of using lineage data instead of individual trajectories for inference is that the uncertainty regarding the initial conditions of the system states (*x*_*d*_(*t*),*D*(*t*),*F*(*t*)) of each cell is greatly reduced, since the prior of each daughter state is based on the posterior of its mother. On the contrary, when the cell trajectories are assumed to be independent, the state of every cell has to be independently initialized according to an assumed prior (described in detail in Additional file 1). Moreover, when the switching rates correspond to mean holding times that exceed the lifetime of a single cell, inference based on a relatively small number of single-cell trajectories will tend to produce biased estimates, as our results show.

## Conclusions

In this work we proposed a parameter inference method for stochastic single-cell dynamics from tree-structured data. More specifically, we considered a class of systems with one or more unobserved states and fluorescent reporter readouts, observed through time-lapse microscopy, which allows tracking individual cells and their progeny over time. Our goal was to estimate the posterior distribution of the unknown system parameters given such readouts. To calculate the likelihood of the data for a given parameter set, the hidden state trajectories had to be integrated out. This marginalization was accomplished with the help of a sequential Monte Carlo method, which recursively computes a sampling-based estimate of this analytically intractable quantity. To sample the system parameter space, we employed an MCMC scheme, which was able to target the correct parameter posterior despite the noisy likelihood estimates. The application of our method to two simple examples showed that it can correctly infer the parameters of interest and approximate their posterior distribution. Our algorithm is currently implemented in C++ and an inference run for the second example presented, consisting of 111,000 MCMC iterations, took about 24 h.

Our inference framework extends to more complex applications in a straightforward manner (for instance larger stochastic chemical reaction networks), although its computational complexity increases with the state and parameter dimensionality. More complex networks will require heavier stochastic simulations, which take a crucial part of the computations carried out in our method. They will also require a larger number of particles to achieve a reasonable accuracy of the SMC-based likelihood estimator. If the latter is too noisy, one may observe slow mixing of the MCMC sampler and in turn poor posterior estimates.

The choice of the MCMC sampler is also a critical issue and depends on the features of the problem at hand, such as the complexity of the target distribution. The Metropolis-Hastings sampler was sufficiently well-suited for the examples presented in this study, but may not be the best choice for every problem. To apply our inference framework on more complex problems, different samplers might need to be employed, with better and more effective convergence and parameter exploration properties. In some cases the model structure might also not be completely known a priori and model selection should be performed to discriminate among several candidate model structures. Bayesian model selection requires the computation of the evidence (marginal likelihood) for each candidate model, which will demand much more powerful and sophisticated samplers than the MCMC sampler presented here. Model selection is, however, beyond the scope of this work.

The inference framework presented here could be very useful in the case of systems where accurate tracking of single-cell dynamics across cell lineages plays an important role. These are, for example, systems involved in stem cell fate decisions, or stochastic phenotype switching in bacteria [8, 10]. In many such cases, stochastic fluctuations of key factors over long timescales and/or stochastic events taking place at cell division create strong mother-daughter and daughter-daughter correlations that play a crucial role in determining the overall behavior of a colony. In such cases, treatment of the measured single-cell trajectories independently from each other will result a large loss of information and biased parameter estimates. We believe that proper incorporation of the population lineage information into the parameter inference problem will thus provide the right framework for treating this type of systems and may reveal important insights into their function.

## Additional files

Additional file 1 Further details about the examples presented. (PDF 6448 kb)

Additional file 2 A ZIP file containing the raw datasets obtained from the MCMC runs presented. (ZIP 449 kb)

## Abbreviations

ABC: Approximate Bayesian Computation
CTMC: Continuous-time Markov chain
MCMC: Markov chain Monte Carlo
SMC: Sequential Monte Carlo
