# Supplementary material for: Parameter inference for stochastic single-cell dynamics from lineage tree data
Source: BMC Syst Biol. 2017 Apr 26;11:52. doi: 10.1186/s12918-017-0425-1 (PMC5406901; doi:10.1186/s12918-017-0425-1)
Supplement: Supplementary file 1 — Further details about the examples presented. (PDF 6448 kb) [file 12918_2017_425_MOESM1_ESM.pdf]

Parameter inference for stochastic single-cell dynamics from lineage tree data - Additional File 1

Irena Kuzmanovska, Andreas Milias-Argeitis, Jan Mikelson, Christoph Zechner and Mustafa Khammash

Inference performed on individual trajectories

To better assess the advantages of our method, we performed parameter inference considering each cell trajectory from the cell lineage to be independent from the others for the second example model presented in the main manuscript. By breaking up the tree structure, we thus treated each cell independently from the rest. In this case, the joint likelihood of the set of trajectories is simply the product of the likelihoods of individual trajectories, given in Eq. 1 below, where individual trajectories are indexed by k.

$$P(\boldsymbol{Y}_{dataset}|\Theta) = \prod_{k=1}^{K} P(\boldsymbol{Y}^k|\Theta) \tag{1}$$

in which the likelihood calculation $P(\boldsymbol{Y}^k|\Theta)$ is done with a classical filtering approach. The rest of the inference method, including the MCMC sampler, was identical with the one used for the lineage data.

Details about the examples presented in the main manuscript

Below we present some details of the synthetic dataset generation, as well as more detailed description about the inference runs presented in the main manuscript.

Data simulation

Table S1: Parameters used in the synthetic data generation

**General**	**Example 1**	**Example 2**
Number of trees<br>Number of generations	1<br>7	1<br>5
Cell division time (min)	30	30
Measurement interval (min)	5	5
**Measurement model related**		
GFP production rates for OFF type α_{OFF} (min^{-1})	0.2	$\mu_{\alpha_{OFF}} = 0.2$
GFP production rates for ON type α_{ON} (min^{-1})	1	$\mu_{\alpha_{ON}} = 1$
GFP maturation rate m (min^{-1})	0.0462	0.0462
GFP dilution rate δ (min^{-1})	0.0261	0.0261
GFP to fluorescence scaling constant c	100	100
Measurement variance σ^2	500	500
Extrinsic variability σ_{ext}	None	0.3

Inference runs

For the inference runs presented in the main manuscript, the initial conditions for the hidden states were drawn as described below. The initial value for the hidden type $x_{d0}^{1,l}(0)$ for the l-th particle was drawn from a uniform distribution over the two possible discrete types (ON and OFF). The initial values for the GFP immature and mature ($D_0^{1,l}(0)$ and $F_0^{1,l}(0)$ respectively) for the l-th particle were drawn from a multivariate normal distribution with mean and covariance corresponding to the sample mean and sample covariance of the initial conditions of all the cells of the simulated tree.

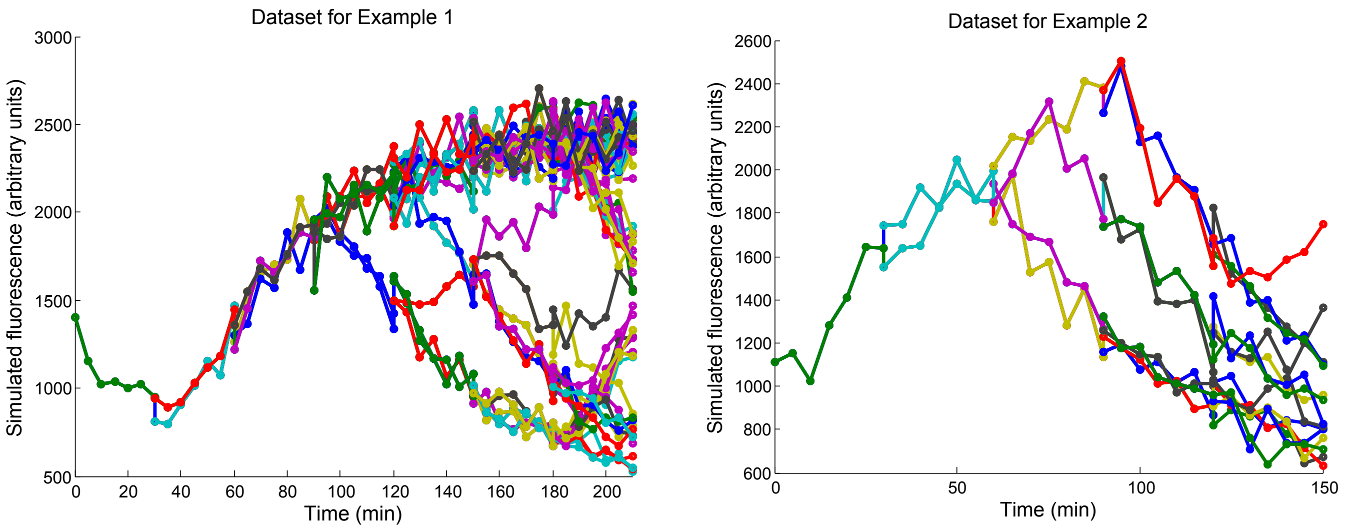

Figure S1: The simulated cell lineage fluorescence used for the inference runs for both examples presented in the main manuscript. The dataset used for the inference in the first example in the main manuscript (left) consisted of a single seven-generation long tree in which the state of the initial mother was of type OFF. The dataset used for the second example (right) consisted of a single five-generation tree in which the state of the initial mother was of type ON. Different colors represent the fluorescence of different cells.

For all of the inference runs presented we used 1000 SMC particles.

The inference run presented in Example 1 was done with 18604 MCMC steps. For the analysis and the posteriors presented in Figure 3 in the main text, the first 2000 samples were discarded. The transition probabilities θ_1 and θ_2 were sampled from the three-dimensional simplex with the help of a Dirichlet distribution $\mathrm{Dir}(\alpha)$, where $\alpha = (\alpha_1, \alpha_2, \alpha_3) = (\theta_1, 2\theta_2, 1 - \theta_1 - 2\theta_2)$ and $\alpha_0 = \sum_i \alpha_i = 100$. The mode of the distribution was located at the current parameter values. The other two parameters θ_3 and θ_4 were sampled independently from a second Dirichlet distribution in a similar fashion.

The tree-based and trajectory-based inference runs presented in Example 2 were done with more than 100000 MCMC steps. In order to ensure that the obtained posterior distributions for this example are not affected by the chain autocorrelation, after discarding the first 15000 samples, we performed thinning of each MCMC chain by subsampling every 200th subsequent sample. Even though the thinned chain showed substantially lower autocorrelations of the samples (Figure S14 and S15), the posteriors obtained with the thinned chain (Figure 5, Figure S12) and the original chain (Figure S13) were visually almost identical. Table S2 summarizes the true value of the parameters, parameter priors and kernel used in each of the runs. All the parameters in these runs (except σ_{ext}) were sampled in $\log_{10}$ scale. The priors for those parameters that were sampled in $\log_{10}$ scale were $\log_{10}$ uniform and as a proposal kernel normal distribution was used, centered on the currently sampled parameter (in $\log_{10}$ scale).

Table S2: Details about some parameters related with the inference run in Example 2 in the main text

Parameter to be inferred	True value	Scale in which parameter was sampled	Prior	Proposal kernel (given currently sampled θ_k)
q_1	0.005	$\log_{10}$	$\log_{10}\mathcal{U}([-6,1])$	$\mathcal{N}(\theta_k, 0.02)$
q_2	0.02	$\log_{10}$	$\log_{10}\mathcal{U}([-6,1])$	$\mathcal{N}(\theta_k, 0.02)$
$\mu_{\alpha_{ON}}$	1	$\log_{10}$	$\log_{10}\mathcal{U}([-6,1])$	$\mathcal{N}(\theta_k, 0.002)$
δ	0.0261	$\log_{10}$	$\log_{10}\mathcal{U}([-6,1])$	$\mathcal{N}(\theta_k, 0.002)$
σ_{ext}	0.3	linear	$\mathcal{U}(0.01, 1)$	$\mathcal{N}(\theta_k, 0.02)$
σ^2	500	$\log_{10}$	$\log_{10}\mathcal{U}([-1,4])$	$\mathcal{N}(\theta_k, 0.02)$

Additional figures and tables for Example 1 and 2

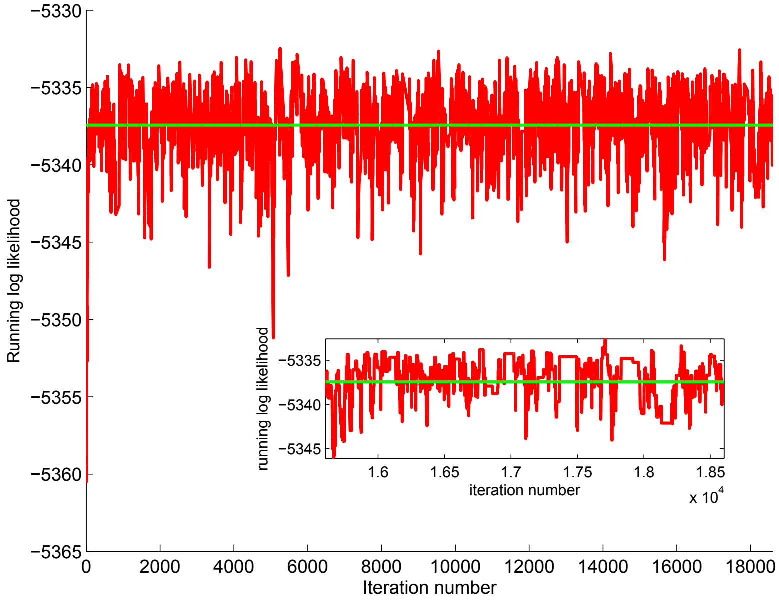

Figure S2: Running log-likelihood vs. number of MCMC iterations (in red) for the inference run presented in Example 1 in the main text. The inlet is a zoom-in of the running log-likelihood of the last 3000 samples to better visualize the convergence. The green line represents the true log-likelihood value (obtained by averaging of 100 log-likelihood calculations with 1000 SMC particles and with the true parameter values).

Table S3: Statistical comparison of the marginal posterior distributions for Example 1 obtained with the exact likelihood calculation method (Figure S8) and our likelihood approximation method (Figure 3). The statistical value in each cell of the table is reported first for the posteriors obtained with the exact likelihood calculation method, followed by those obtained with our approximate likelihood calculation method.

	**posterior mean**	**posterior variance**	**posterior median**
θ_1	0.6287 / 0.6214	0.0129 / 0.0129	0.6362 / 0.6295
θ_2	0.0568 / 0.0605	0.0013 / 0.0018	0.0507 / 0.0501
θ_3	0.0953 / 0.0945	0.0022 / 0.0021	0.0860 / 0.0877
θ_4	0.0712 / 0.0722	0.0007 / 0.0007	0.0678 / 0.0704

Table S4: Tree-based inference: The correlation coefficients corresponding to the scatter plots in Figure S16.

Example 2						
	$log_{10}(q_1)$	$log_{10}(q_2)$	$log_{10}(\mu_{\alpha_{ON}})$	$log_{10}(\delta)$	σ_{ext}	$log_{10}(\sigma^2)$
$log_{10}(q_1)$	1.00	0.12	0.10	-0.07	-0.09	0.07
$log_{10}(q_2)$		1.00	0.19	-0.002	0.38	-0.08
$log_{10}(\mu_{\alpha_{ON}})$			1.00	-0.24	-0.28	0.04
$log_{10}(\delta)$				1.00	0.93	-0.25
σ_{ext}					1.00	-0.25
$log_{10}(\sigma^2)$						1.00

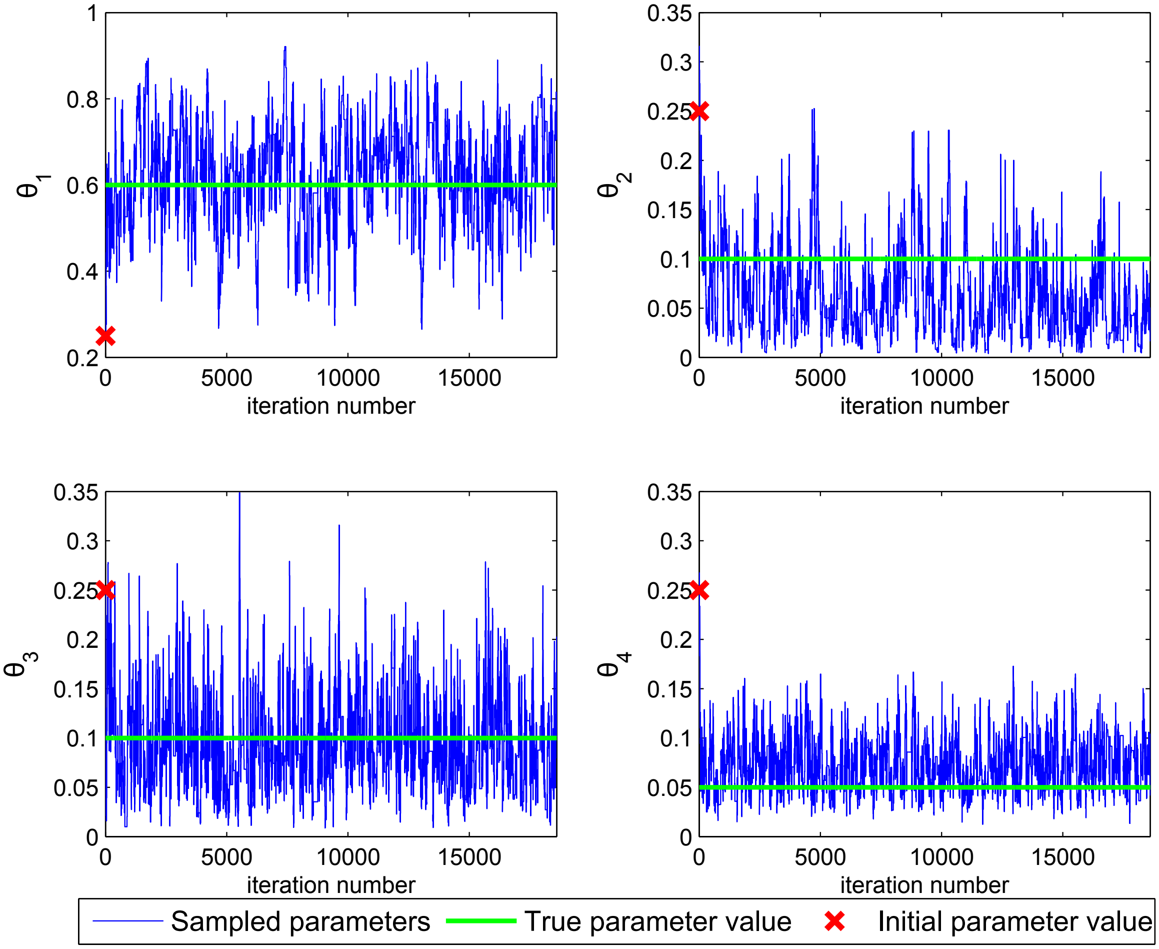

Figure S3: Movement of the MCMC chain in each dimension of the parameter space for the inference run presented in Example 1 in the main text. The green line indicates the true parameter value. The red cross indicates the parameter value where the chain is initialized.

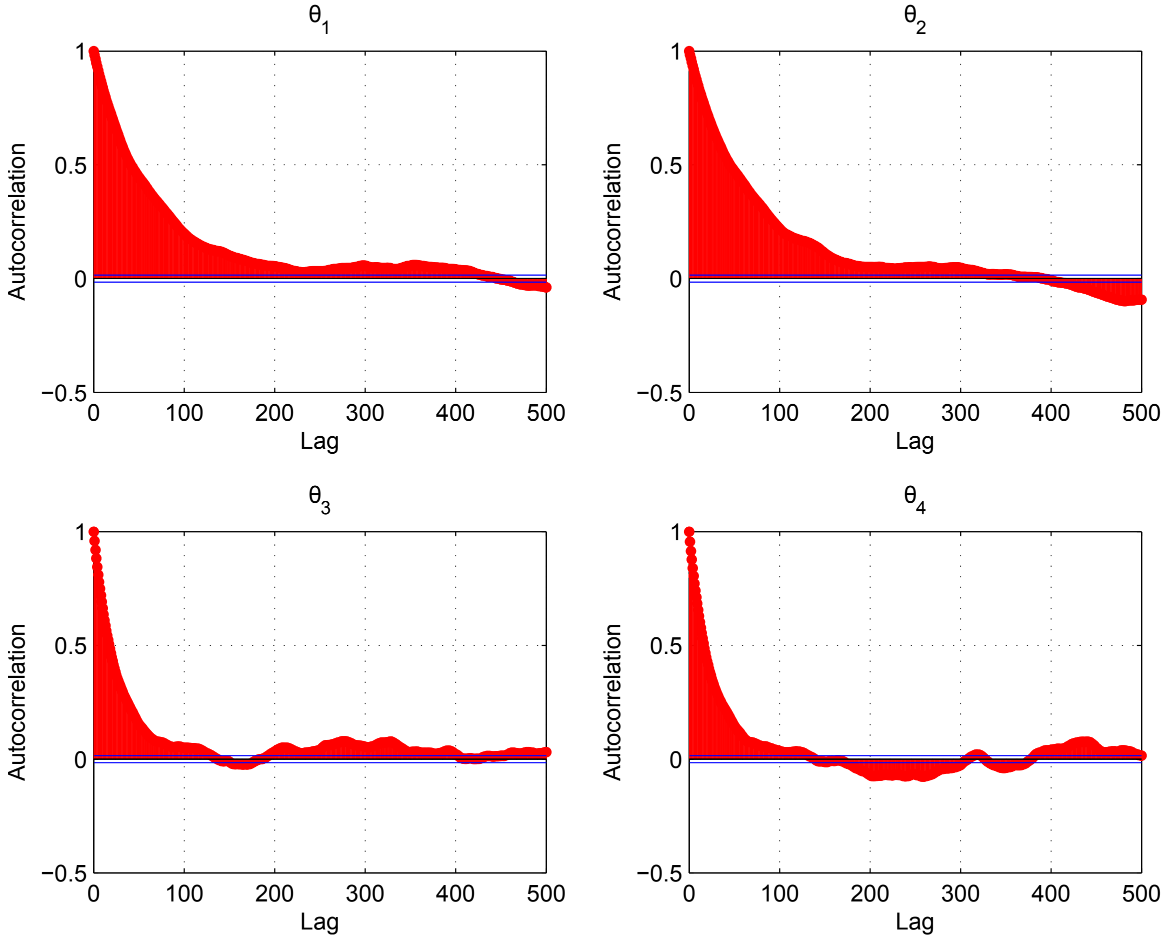

Figure S4: Autocorrelation of the sampled parameters presented in the Figure 3 of the main manuscript, for Example 1. A burn-in of the initial 2000 samples has been discarded.

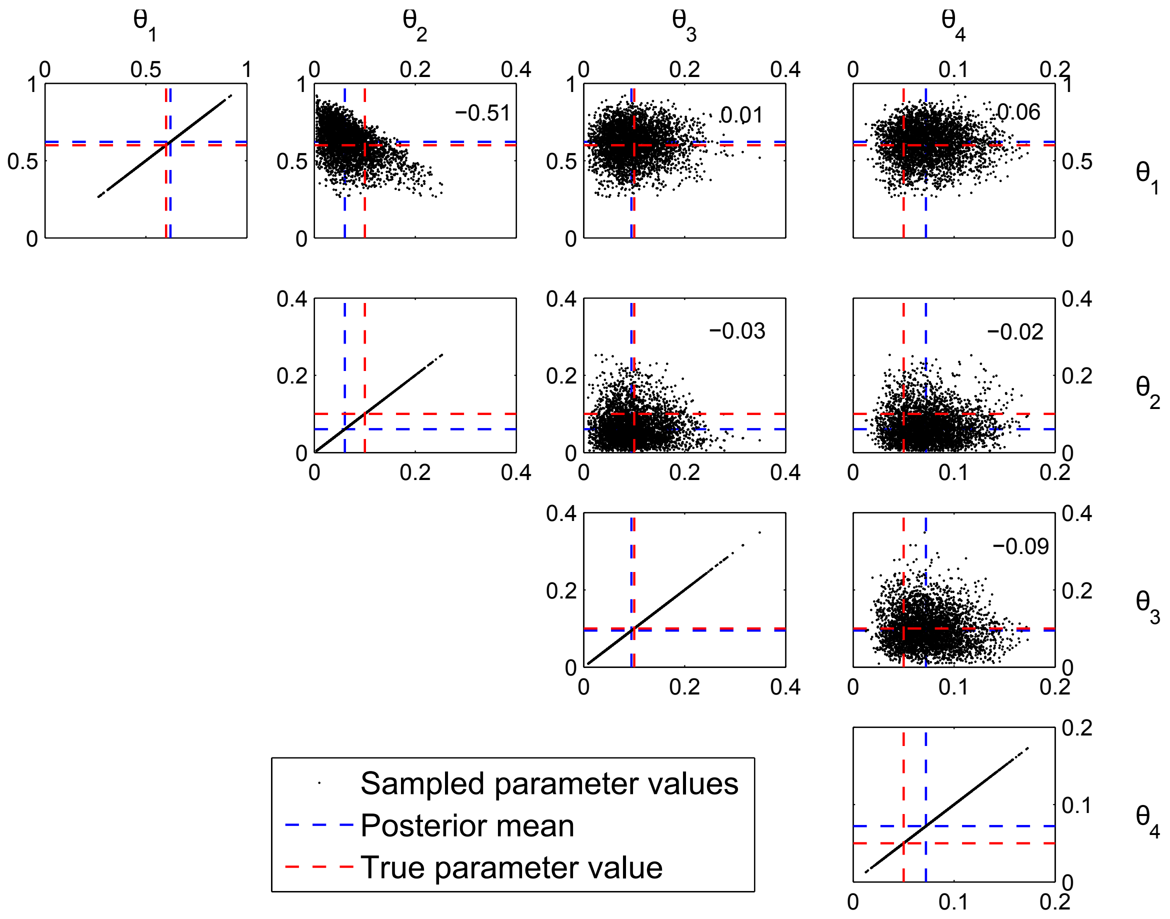

Figure S5: Pairwise scatter plots of the sampled parameters presented in the Figure 3 of the main text, for Example 1. The coefficients of correlation for each scatter plot are given in the top right corner of the corresponding plot.

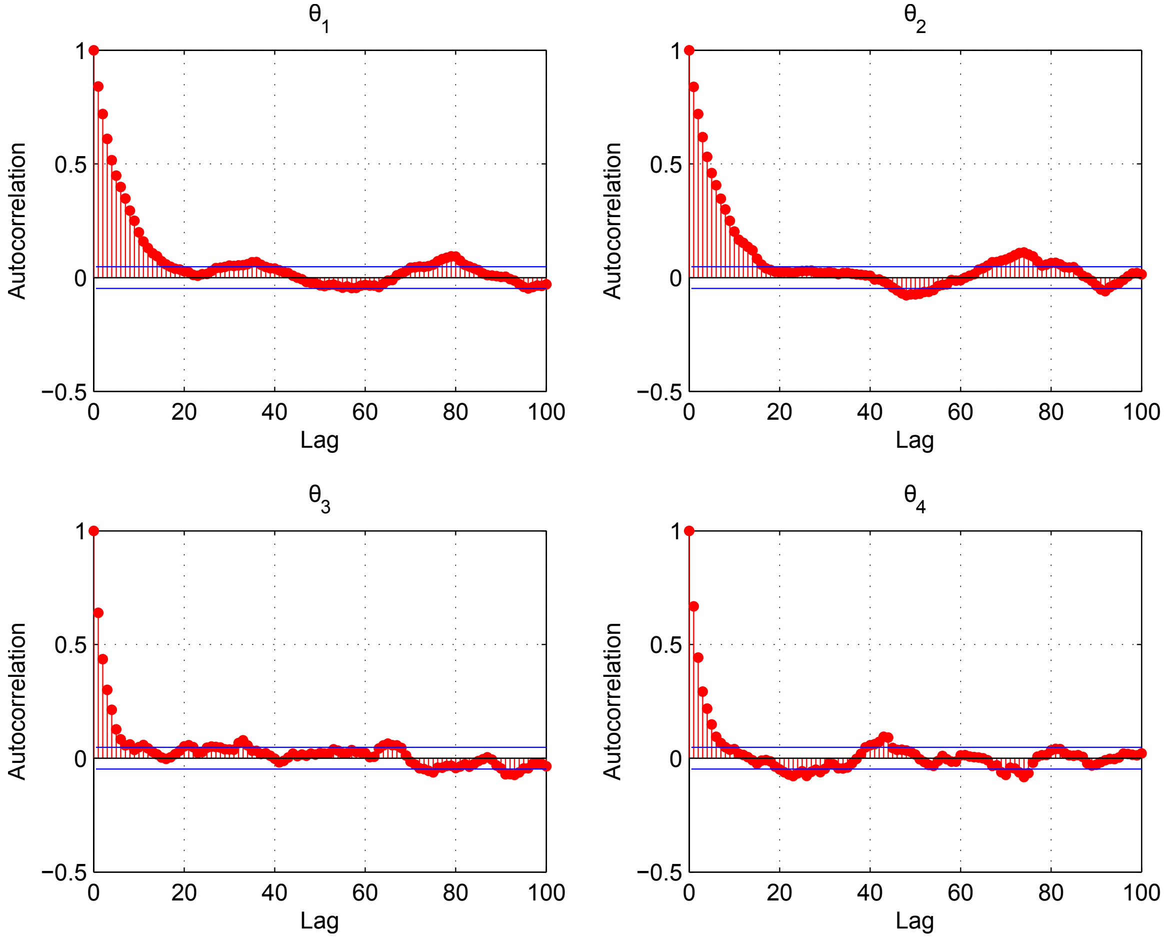

Figure S6: Autocorrelation of the thinned MCMC chain from Example 1. After the initial 1000 samples have been discarded from the original chain, a new chain was created by subsampling every 10th sample from the remainings of the original chain. The autocorrelation of this new thinned chain drops quicker than the autocorrelation of the original chain (Figure S4).

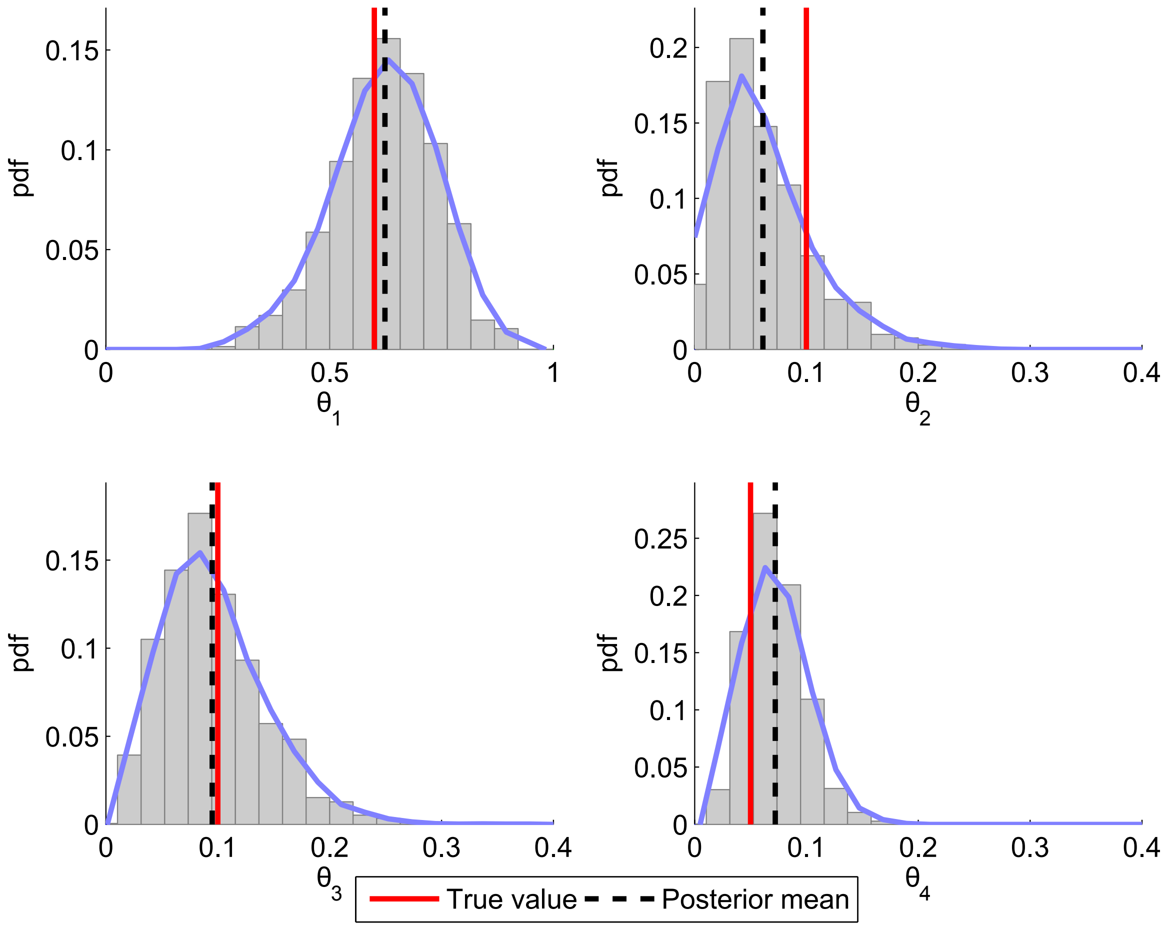

Figure S7: Posterior distributions obtained from the thinned MCMC chain from Example 1, consisting of total of 1761 samples.

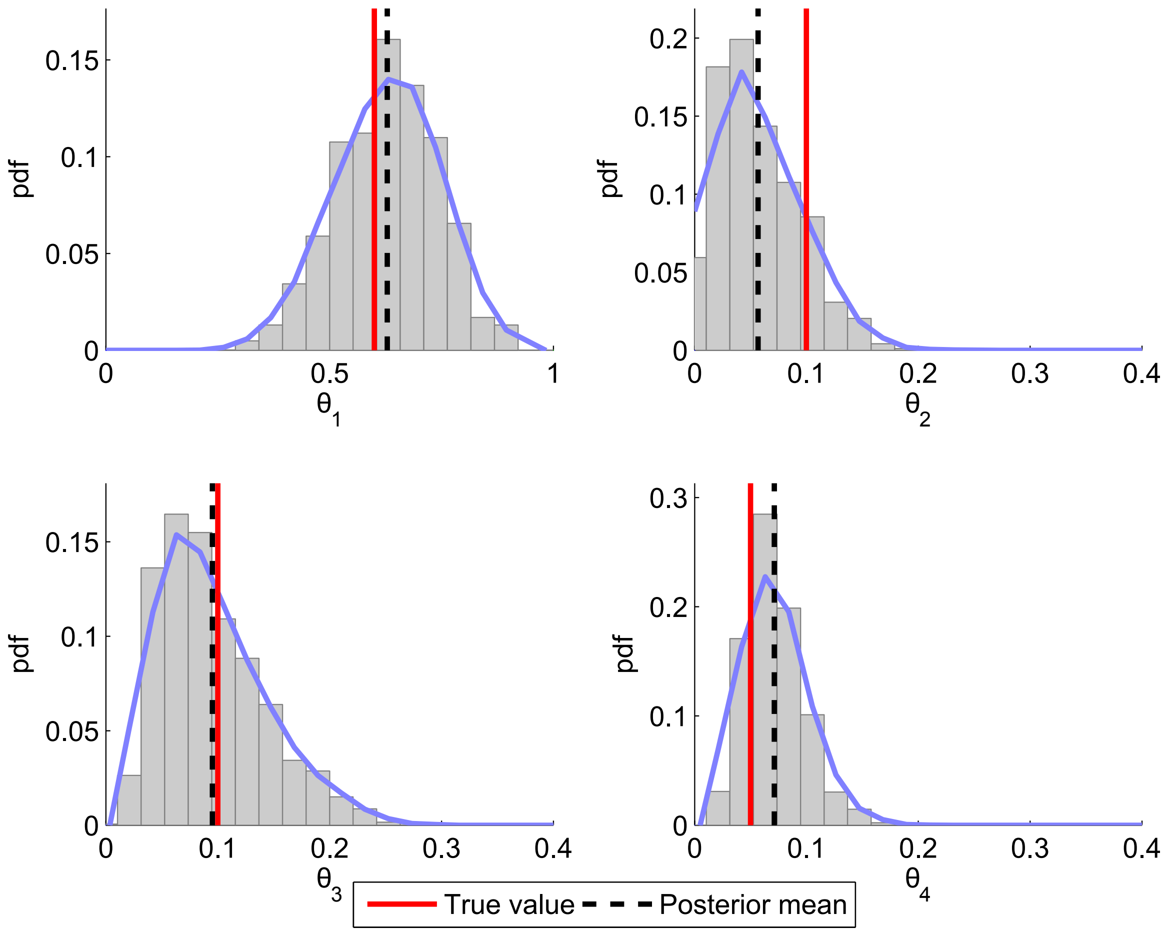

Figure S8: Posterior distributions obtained with inference in which the exact likelihood calculation was used. The posteriors are visually identical to the posteriors in Figure 3 in the main manuscript, which indicates that the employment of our simplifying assumption in the likelihood calculation does not create significant bias in the inference procedure.

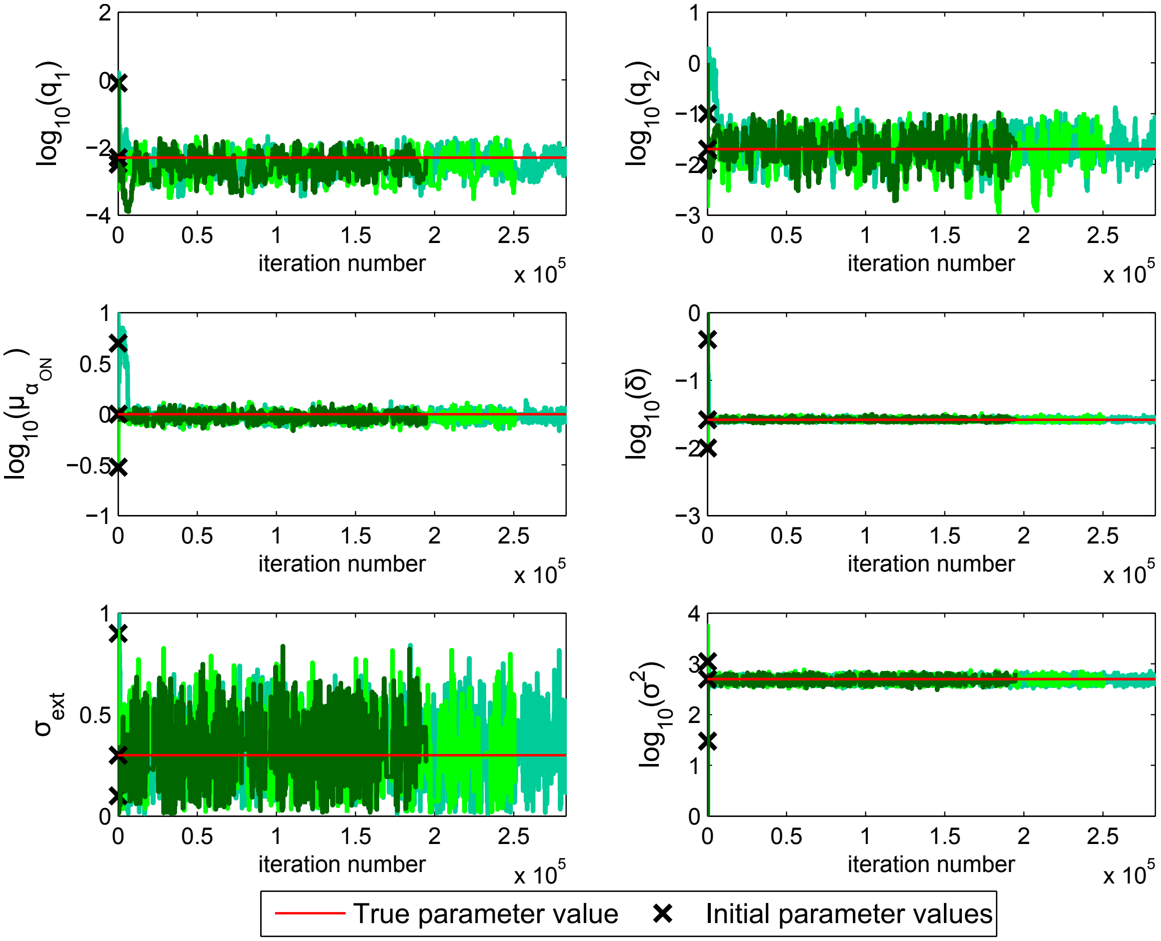

Figure S9: Tree-based inference: Chain movement of three independent tree-based MCMC chains for Example 2 in the main text, initialized from different initial conditions (black crosses). Each chain is colored in different shade of green. One of the chains was used to obtain the tree-based posteriors on Figure 5 in the main text. The true parameter value is indicated with red line. One can notice that regardless the initial conditions, the chains always converge to the same region in the parameter space.

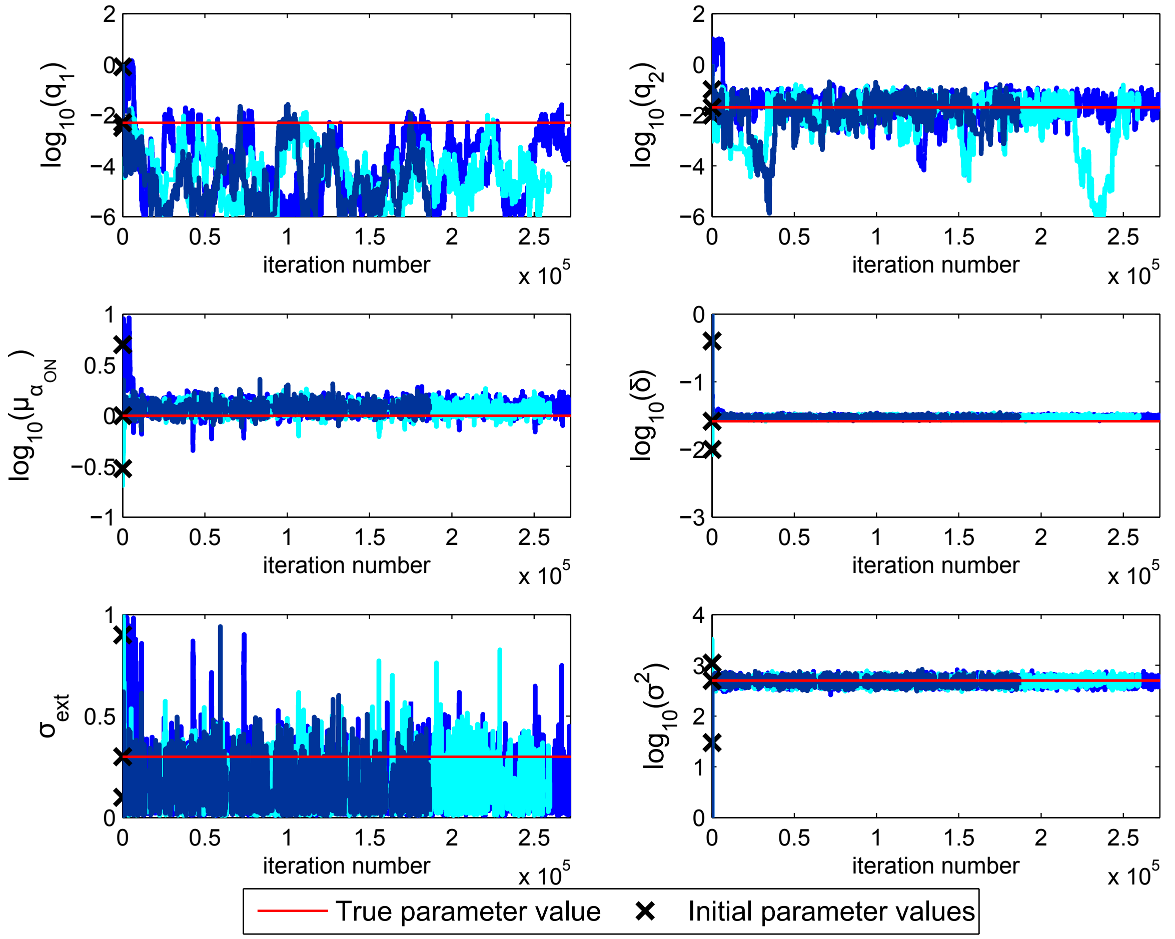

Figure S10: Trajectory-based inference: Chain movement of three independent trajectory-based MCMC chains for Example 2 in the main text, initialized from different initial conditions (black crosses). Each chain is colored in different shade of blue. One of the chains was used to obtain the trajectory-based posteriors on Figure 5 in the main text. The true parameter value is indicated with red line. Regardless the initial conditions, the chains always converge around the same region in the parameter space.

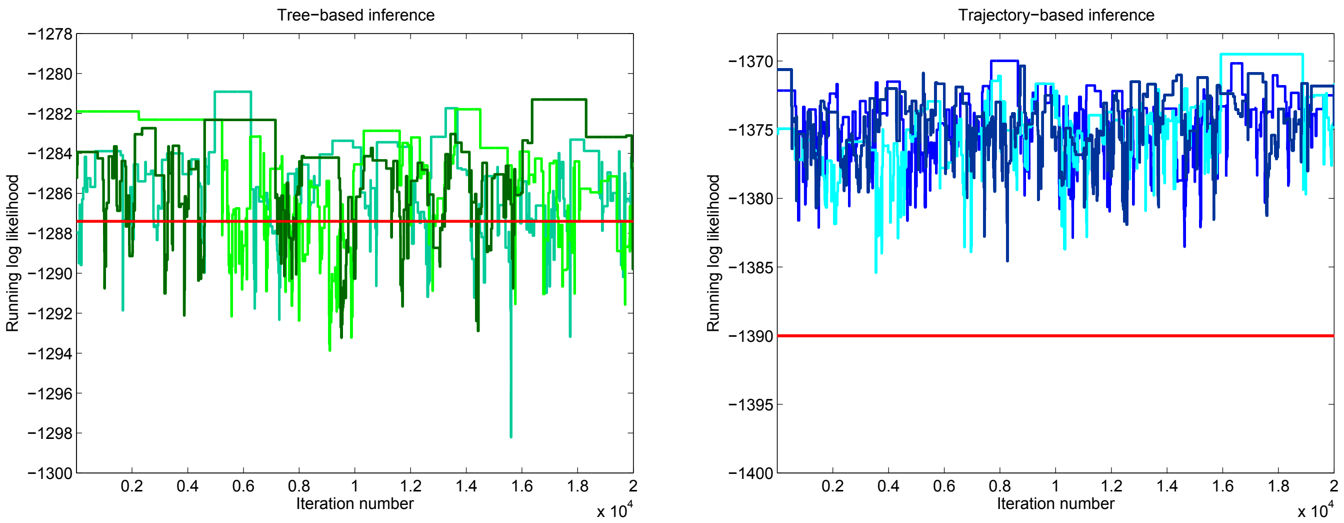

Figure S11: Running log-likelihood for the independent tree-based (left) and the independent trajectory-based (right) MCMC chains, of the last 20000 iterations of each chain. The iteration number on the x-axis is relative to the portion of the chain considered. The red line indicates the log-likelihood value obtained as an average of 500 log-likelihood calculations with the appropriate method when the true parameters were used.

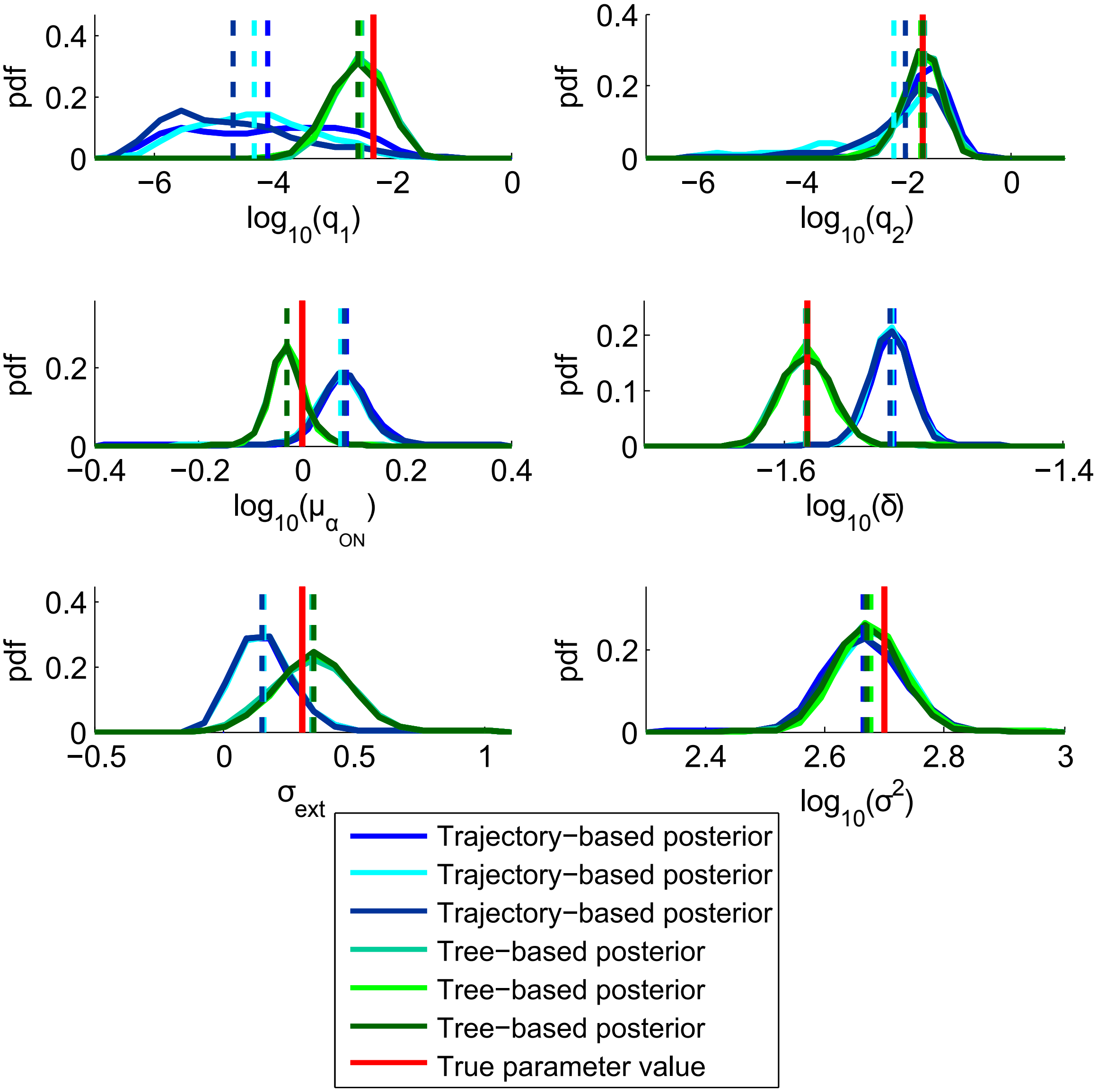

Figure S12: Posterior distributions of the unknown parameters presented in Example 2 in the main text, obtained both with tree-based and trajectory-based inference. The posterior distributions from the three independent thinned MCMC chains for each type of inference are overlayed. The distributions obtained from one of the three chains for each inference type are also shown in Figure 5 in the main text. The red bars are positioned at the true parameter values (i.e. the ones used for data generation), while the dashed lines indicate the estimated posterior means. The curves are obtained by smoothing of the normalized histograms of the MCMC samples. One can clearly observe the bias in the parameter estimates when trajectory-based inference was used.

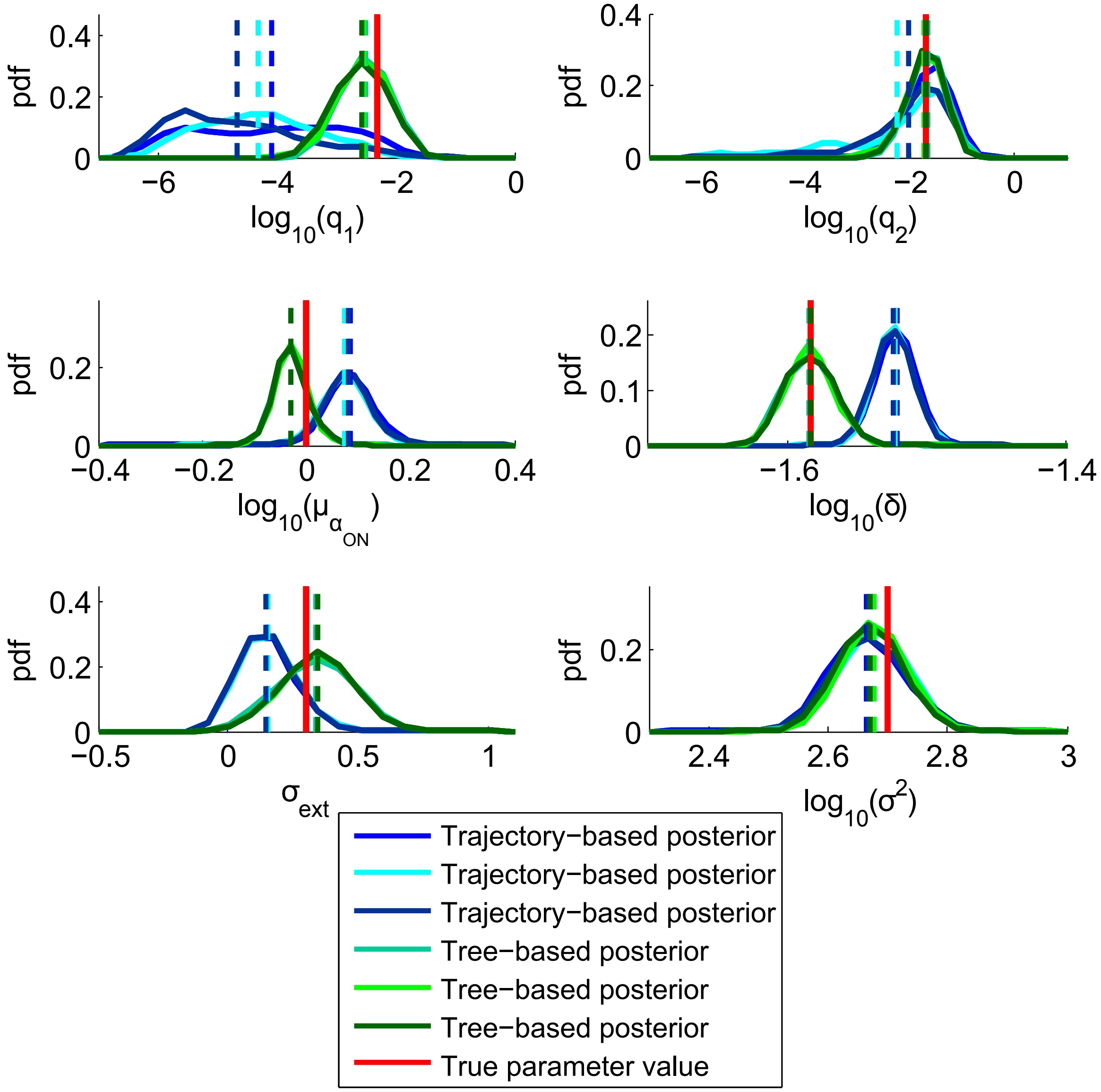

Figure S13: The posterior distributions which are given in Figure S12, but obtained from the original, unthinned MCMC chains (Figures S9 and S10), after the first 15000 steps had been discarded. The distributions obtained with the original and the thinned chains are visually identical.

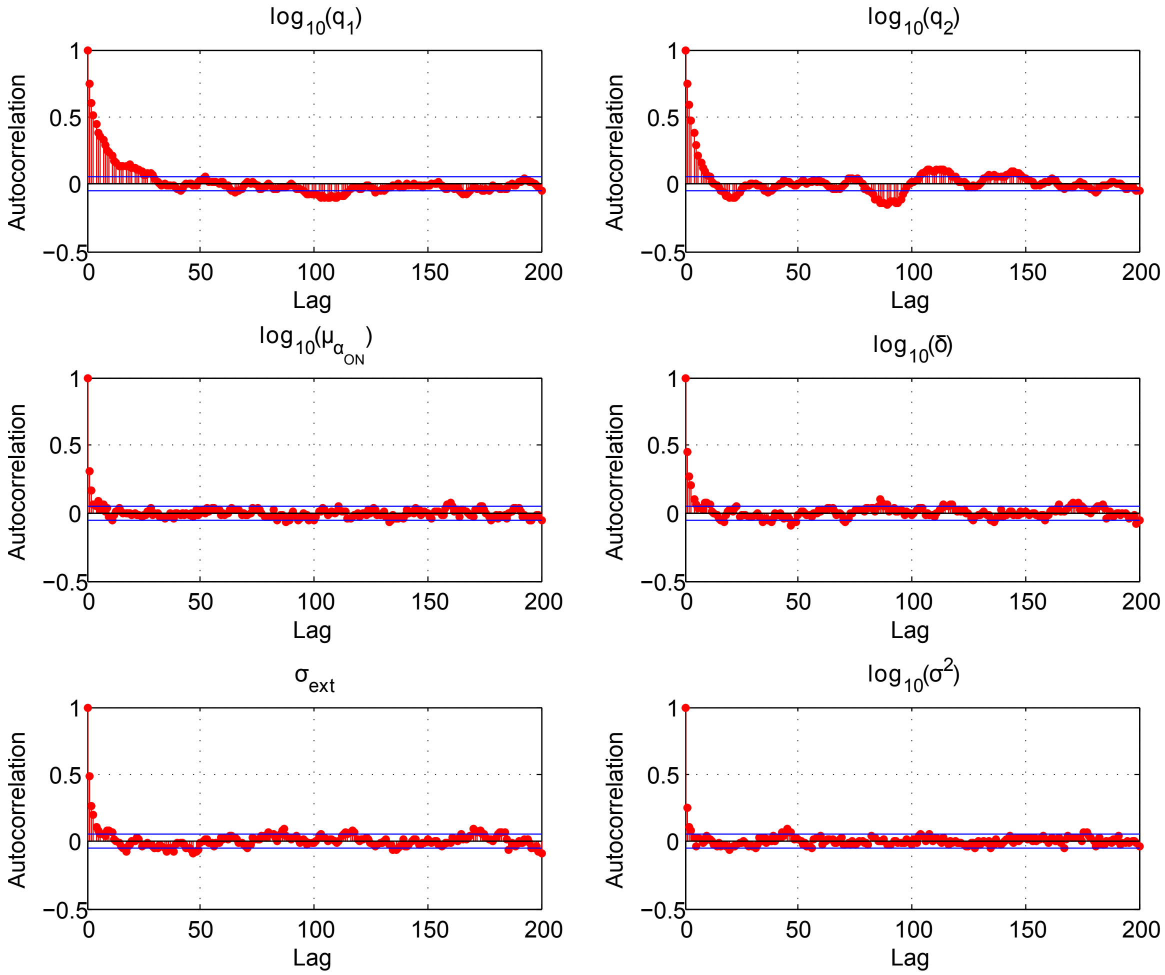

Figure S14: Tree-based inference: Sample autocorrelation of the thinned MCMC chain which was used to obtain the tree-based posterior distributions in Figure 5 in the main text.

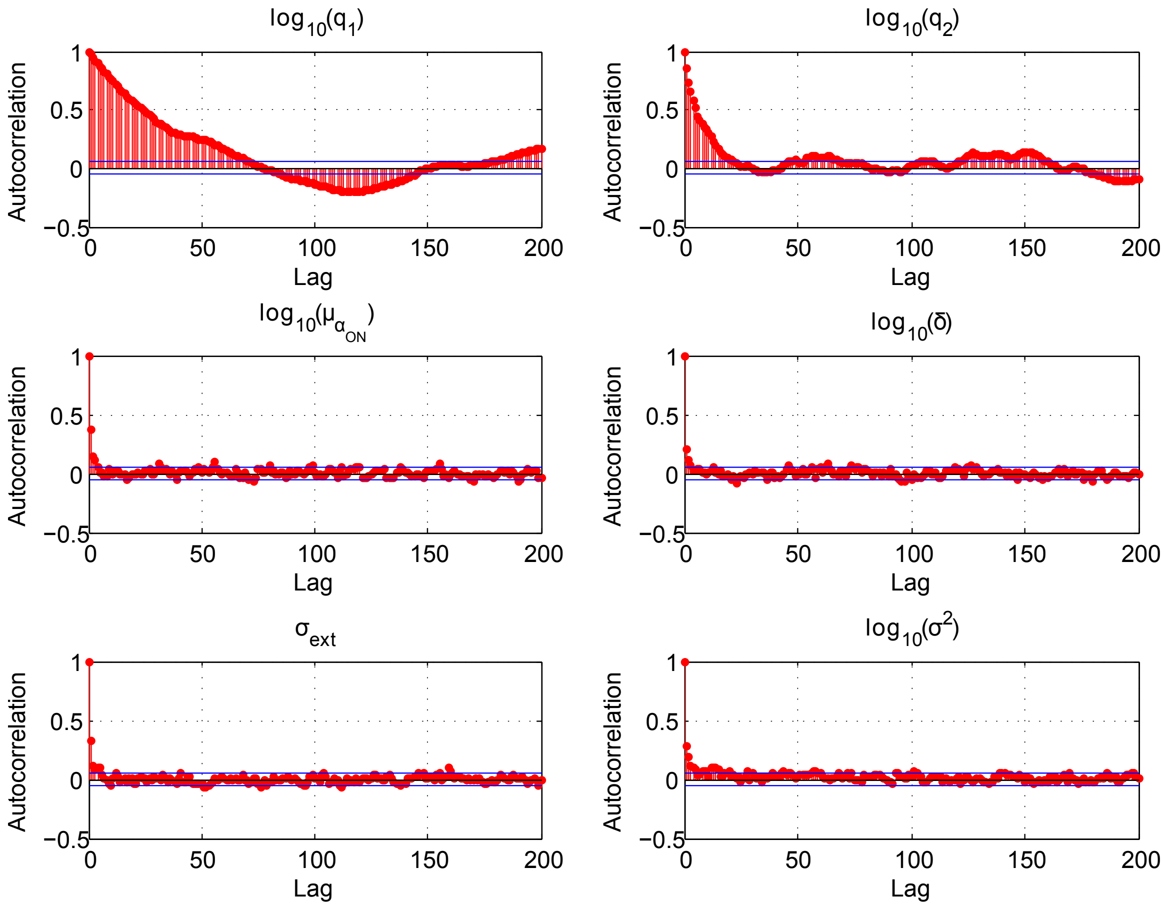

Figure S15: Trajectory-based inference: Sample autocorrelation of the thinned MCMC chain which was used to obtain the trajectory-based posterior distributions in Figure 5 in the main text.

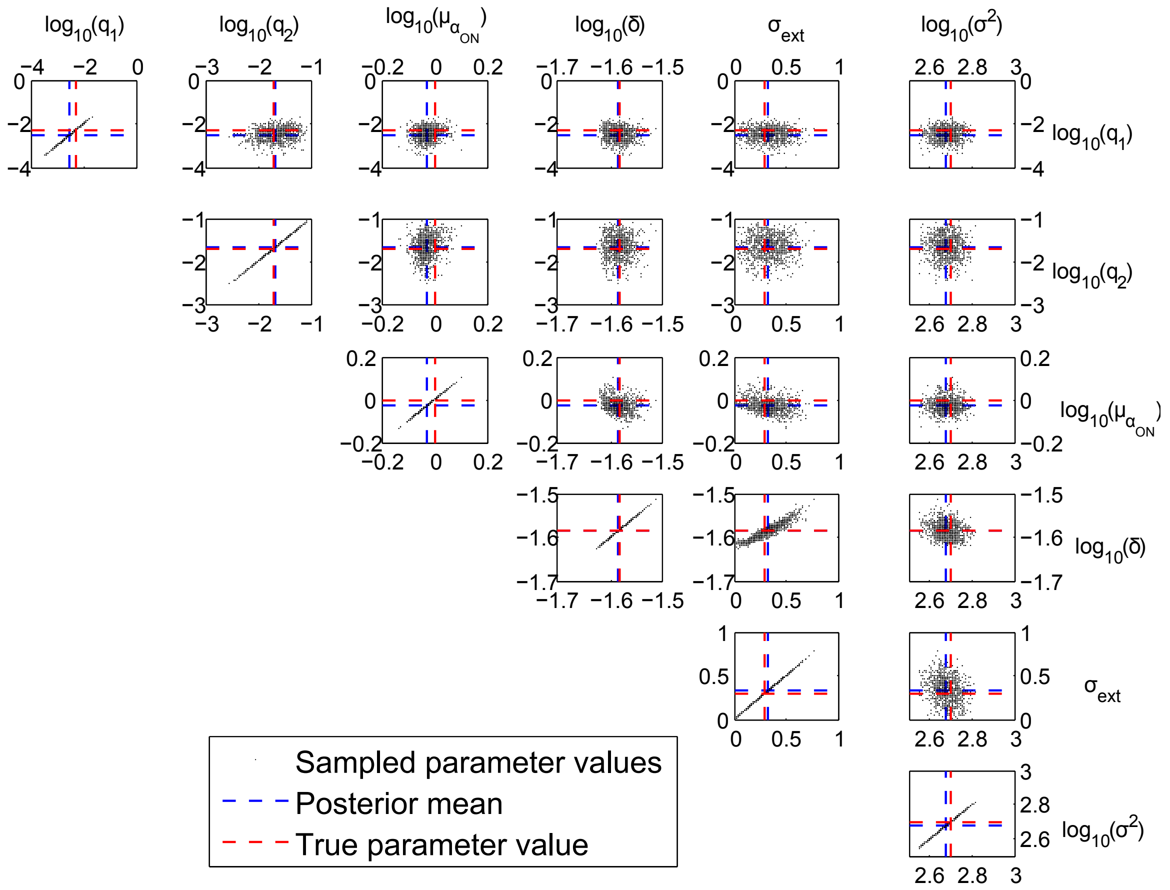

Figure S16: Tree-based inference: Pairwise scatter plots of the sampled parameters used to obtain the tree-based posteriors in Figure 5 in the main text. The correlation coefficients corresponding to each plot are given in Table S4

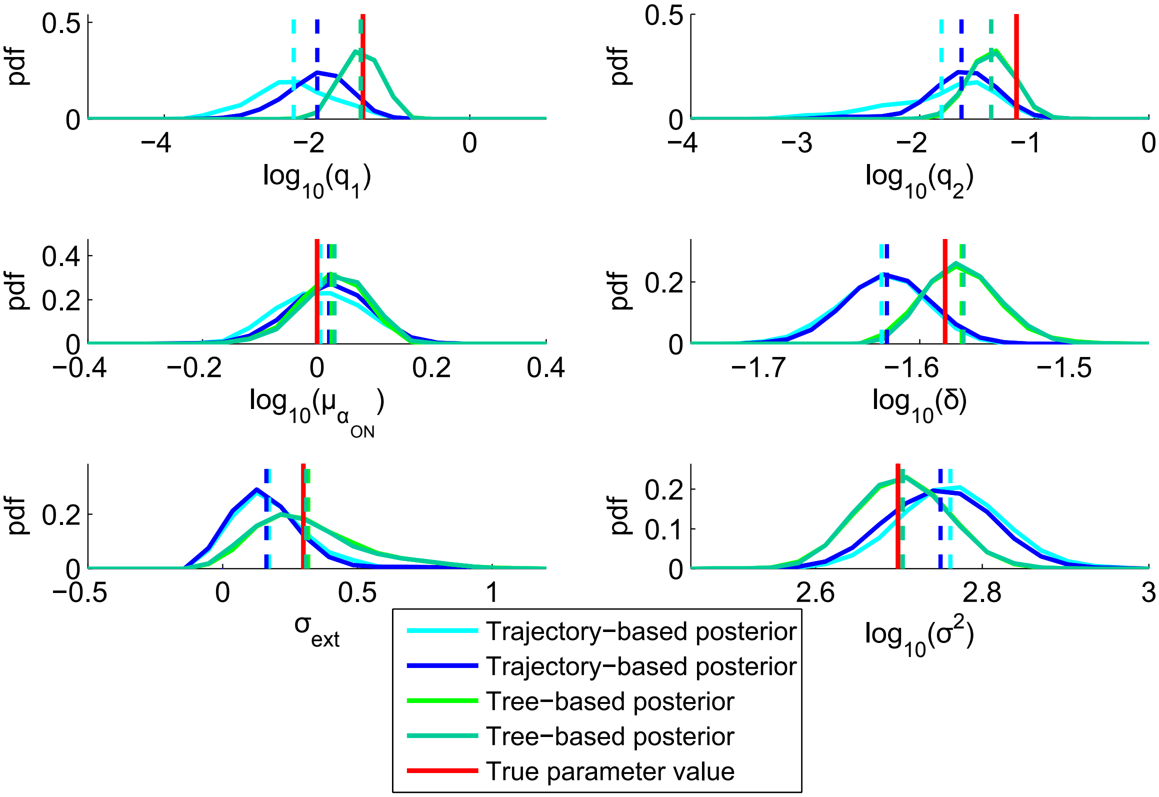

Figure S17: Tree-based and trajectory-based posterior distributions for another Example 2 dataset (not described in the main text), for which different values for the switching rates were used. The value for q_1 was 0.04 and the value for q_2 was 0.07. The rest of the parameter values were as presented in Table S2. The inference runs in this case were done similarly as for the example presented in the main text and with parameters described in Table S2, except the proposal kernel for q_1, q_2 and σ_{ext} had variance which is one order of magnitude lower than the corresponding value in Table S2. No thinning was performed for the posteriors in this figure. We also obtained posterior distributions after chain thinning (not shown), but they were visually identical to the posterior distributions presented here.
