# Supplementary material for: Parameter inference for stochastic single-cell dynamics from lineage tree data
Source: BMC Syst Biol. 2017 Apr 26;11:52. doi: 10.1186/s12918-017-0425-1 (PMC5406901; doi:10.1186/s12918-017-0425-1)
Supplement: Supplementary file 2 — A ZIP file containing the raw datasets obtained from the MCMC runs presented. (ZIP 449 kb) [file 12918_2017_425_MOESM2_ESM.zip › Additional File 2/Description.pdf]

Parameter inference for stochastic single-cell dynamics from lineage tree data - Additional File 2

Irena Kuzmanovska, Andreas Milias-Argeitis, Jan Mikelson, Christoph Zechner and Mustafa Khammash

In the current folder the raw datasets obtained from the MCMC runs are provided, upon which the posterior distributions in Figure 3 and Figure 5 in the main text and Figure S8 in the Additional File 1 are based. A single MCMC run produces two files (one containing the word '*parameters*' and the other '*likelihoods*' in their name). The former file contains all accepted parameter sets (each line corresponding to a different parameter set). The '*likelihoods*' file consists of two columns. The right column contains the log-likelihood values of all the accepted parameter sets, given in the same line number in the '*parameters*' file. The value in the left column indicates how many iterations have passed since the last accepted parameter set. Note that if during a MCMC iteration no new parameter set has been accepted, the parameter set from the previous iteration is appended to the chain. For a clearer idea please refer to Algorithm 2 in the main text. In summary, a parameter set in the nth line in the '*parameters*' file is associated with the log-likelihood value in the right column of the nth line in the '*likelihoods*' file. The value in the left column of the $n+1^{th}$ line in the same file indicates how many consecutive iterations this parameter set appears in the chain, before the parameter set in line $n+1$ in the '*parameters*' file has been accepted.

The following files are provided:

File name	Description
Example1_mcmc_parameters.txt	The 'parameters' file associated with the posteriors in Fig. 3
Example1_mcmc_likelihoods.txt	The 'likelihoods' file associated with the posteriors in Fig. 3
Example1_mcmc_parameters_exact.txt	The 'parameters' file associated with the posteriors in Fig. S8
Example1_mcmc_likelihoods_exact.txt	The 'likelihoods' file associated with the posteriors in Fig. S8
Example2_mcmc_parameters_tree.txt	The 'parameters' file associated with the tree-based posteriors in Fig. 5
Example2_mcmc_likelihoods_tree.txt	The 'likelihoods' file associated with the tree-based posteriors in Fig. 5
Example2_mcmc_parameters_traj.txt	The 'parameters' file associated with the trajectory-based posteriors in Fig. 5
Example2_mcmc_likelihoods_traj.txt	The 'likelihoods' file associated with the trajectory-based posteriors in Fig. 5
